# Proteomic signatures of vaccine-induced immunity in teleost fish: from antigen discovery to correlates of protection

**DOI:** 10.3389/fimmu.2026.1965383

**Published:** 2026-10-26

**Authors:** Shreesha Sadashiva Rao, T. S. Keshava Prasad

**Affiliations:** Nitte (Deemed to be University), Center for Omics & Systems Medicine (C-OSM), Department of Biochemistry, KS. Hegde Medical Academy (KSHEMA), Mangalore, Karnataka, India

**Keywords:** biomarker, correlates of protection, mass spectrometry, proteomics, teleost vaccinology

## Abstract

Fish-vaccine efficacy in teleosts is still established primarily through pathogen-challenge experiments, which determine whether a formulation protects but provide limited insight into the antigens and immune mechanisms responsible. Proteomics addresses part of this limitation by measuring pathogen proteins and host responses induced by vaccination. This critical narrative review evaluates pathogen-focused and host-response proteomics from antigen discovery through biomarker validation and translational decision-making. We assess quantitative mass spectrometry, immunoproteomics, surfaceome and secretome profiling, and their integration with reverse and systems vaccinology. Across the limited teleost evidence base, vaccination-associated changes involve complement and acute-phase pathways, antigen processing, redox regulation, hemostasis, tissue repair and exosome-associated antigen presentation. Cross-study replication remains weak, and the reported proteins largely represent study-specific vaccine-responsive profiles. More importantly, most studies do not test whether pre-challenge signatures predict subsequent survival, pathogen control or tissue protection. Within the literature reviewed here, no candidate proteomic signature has yet been prospectively replicated in an independent teleost vaccination cohort; therefore, none currently meets the requirements of a validated, transferable correlate of protection. The main obstacles include heterogeneous study designs, incomplete reference proteomes, teleost-specific protein-inference challenges, small cohorts, limited longitudinal sampling and scarce external validation. We argue that the principal bottleneck is now study design and validation rather than mass-spectrometric capability alone. We therefore outline a development sequence that begins with infection relevant antigen selection and proceeds through longitudinal profiling, targeted verification, predictive modeling, independent validation and field evaluation. This framework separates vaccine responsiveness, protection association, prediction and mechanism, using proteomics to support vaccine-development decisions while retaining challenge testing.

## 1 Introduction

Aquaculture is a rapidly growing food-production sector that contributes substantially to food security and animal-protein supply. However, production intensification has increased the impact of bacterial, viral and parasitic diseases on fish welfare and production (1–4). Vaccination is an important component of disease control because it improves fish health and can reduce antimicrobial use (5). Most commercial fish vaccines are based on inactivated or attenuated pathogens. Many were developed empirically, with limited understanding of the mechanisms responsible for protection (6, 7). These empirical approaches have produced several effective bacterial vaccines, but they provide limited information about the antigens and immune pathways that mediate protection (8). Candidate antigens are commonly prioritized according to abundance, surface exposure or known virulence functions and then tested in vaccination and challenge experiments (7, 9). Achieving consistent protection across teleost species remains challenging because vaccine responses vary with host genetics, pathogen diversity, vaccine platform and environmental conditions (10, 11). Consequently, many economically important aquatic pathogens still lack effective commercial vaccines (2). Better methods are therefore needed to prioritize protective antigens before extensive *in vivo* testing (6, 12).

Fish-vaccine efficacy is commonly evaluated using relative percent survival, antibody titres, histopathology and transcriptional responses (6, 13). Although these measures provide valuable information on vaccine performance, they offer limited mechanistic insight into the molecular processes that underlie protective immunity (14). High-resolution mass spectrometry enables the direct quantification of proteins involved in immune recognition, antigen processing, complement pathways, intracellular signaling and effector responses (15, 16). Because protein abundance is influenced by translation, turnover and post-translational regulation, proteomics provides information that cannot be inferred reliably from transcript abundance alone (17). Although proteomics has become an established tool in fish immunology, its application to vaccinology remains fragmented. Differences in species, pathogens, tissues, vaccine platforms and analytical workflows limit comparisons among studies (18, 19). As a result, few candidate biomarkers have been independently validated, and no broadly validated proteomic correlate of protection has yet been demonstrated (20, 21). Addressing this limitation may support more predictive and mechanistically informed vaccine development.

This review examines proteomics across teleost vaccine development, from pathogen-antigen discovery and host-response profiling to candidate correlates of protection and translational biomarkers. We evaluated the strength of the available evidence, the barriers to cross study comparison and biomarker validation, and the role of proteomics in teleost systems vaccinology. Particular attention is given to distinguishing vaccine-responsive proteins from protection-associated, predictive and mechanistic correlates. Finally, we propose a proteomics-enabled framework that connects infection-relevant antigen prioritization with host-response profiling, outcome-linked biomarker discovery, analytical verification, independent validation and practical vaccine-development decisions.

## 2 Literature search and critical narrative synthesis

This critical narrative review evaluates proteomics across the teleost vaccine-development pipeline, from pathogen-antigen discovery and vaccine-induced host responses to candidate correlates of protection. Structured searches were conducted in PubMed, Scopus and Web of Science Core Collection on 30 June 2026, without a lower publication-date restriction and with searches limited to English-language publications (**Table 1**). Google Scholar and SciSpace supported supplementary literature discovery. Database searches combined teleost host terms with vaccination and proteomic-technology terms. Supplementary queries addressed antigen discovery and protection-associated biomarkers. Database-specific search strings and the numbers of records exported are provided in **Table 2**.

**Table 1:** Literature search framework and execution details.

Source	Role	Conceptualization	Documentation note
Review design	Critical narrative review using structured literature searches and evidence classification	Not a systematic review or meta-analysis	Report complete study inventories in **Supplementary Tables S1**, **S2**
PubMed	Formal bibliographic search	Teleost vaccination proteomics, antigen discovery and host-response biomarkers	Contain three queries (**Table 2**) (n=No restriction on the number of records retrieved),
Scopus	Formal bibliographic search	Title, abstract and keyword fields	Contain three queries (**Table 2**) (n=20)
Web of Science Core Collection	Formal bibliographic search	Topic fields	Contain three queries (**Table 2**) (n=50)
Google Scholar and SciSpace	Supplementary discovery and citation-linked searching	Broad phrase searches and cited-by searching	A follow-up quire during manuscript writing or either a formal search (**Supplementary Table S3**) or citations of the interesting research articles.
Backward and forward citation searching	Supplementary identification	Reference lists and cited-by records of included studies and key reviews	Follow-up quire for a follow-up during writing, by either a formal search (**Supplementary Table S3**) or citations of the interesting research articles.

Structured searches were conducted in PubMed, Scopus and Web of Science Core Collection on 30 June 2026, without a lower publication-date restriction. Searches were limited to English-language publications.

**Table 2:** Database-specific search strategies and record counts for searches conducted on 30 June 2026.

ID	Database	Purpose	Exact search string	Records exported
P1	PubMed	Core vaccination-proteomics search	("Fishes"[Mesh] OR fish[Title/Abstract] OR fishes[Title/Abstract] OR teleost*[Title/Abstract] OR aquacultur*[Title/Abstract]) AND ("Vaccin"[Mesh] OR vaccine*[Title/Abstract] OR immuniz*[Title/Abstract] OR immunis*[Title/Abstract] OR bacterin*[Title/Abstract]) AND ("Proteomics"[Mesh] OR proteome*[Title/Abstract] OR immunoproteom*[Title/Abstract] OR surfaceome*[Title/Abstract] OR secretome*[Title/Abstract] OR "mass spectrometry"[Title/Abstract] OR "LC-MS/MS"[Title/Abstract] OR iTRAQ[Title/Abstract] OR TMT[Title/Abstract] OR DIA[Title/Abstract] OR "data-independent acquisition"[Title/Abstract])	[n = 193]
P2	PubMed	Antigen-discovery supplement	("Fishes"[Mesh] OR fish[Title/Abstract] OR fishes[Title/Abstract] OR teleost*[Title/Abstract] OR aquacultur*[Title/Abstract]) AND ("Proteomics"[Mesh] OR proteom*[Title/Abstract] OR immunoproteom*[Title/Abstract] OR surfaceom*[Title/Abstract] OR secretom*[Title/Abstract] OR "mass spectrometry"[Title/Abstract] OR "LC-MS/MS"[Title/Abstract] OR iTRAQ[Title/Abstract] OR TMT[Title/Abstract] OR DIA[Title/Abstract] OR "data-independent acquisition"[Title/Abstract]) AND (antigen*[Title/Abstract] OR immunogen*[Title/Abstract] OR "vaccine candidate"[Title/Abstract] OR "vaccine candidates"[Title/Abstract])	[n = 246]
P3	PubMed	Biomarker/outcome supplement	("Fishes"[Mesh] OR fish[Title/Abstract] OR fishes[Title/Abstract] OR teleost*[Title/Abstract] OR aquacultur*[Title/Abstract]) AND ("Vaccines"[Mesh] OR vaccin*[Title/Abstract] OR immuniz*[Title/Abstract] OR immunise*[Title/Abstract] OR bacterin*[Title/Abstract]) AND ("Proteomics"[Mesh] OR proteome*[Title/Abstract] OR immunoproteome*[Title/Abstract] OR surfaceom*[Title/Abstract] OR secretom*[Title/Abstract] OR "mass spectrometry"[Title/Abstract] OR "LC-MS/MS"[Title/Abstract] OR iTRAQ[Title/Abstract] OR TMT[Title/Abstract] OR DIA[Title/Abstract] OR "data-independent acquisition"[Title/Abstract]) AND (biomarker*[Title/Abstract] OR signature*[Title/Abstract] OR predict*[Title/Abstract] OR "correlate of protection"[Title/Abstract] OR "correlates of protection"[Title/Abstract] OR survival[Title/Abstract] OR challenge[Title/Abstract])	[n = 85]
S1	Scopus	Core vaccination-proteomics search	TITLE-ABS-KEY (fish OR fishes OR teleost* OR aquacultur*) AND TITLE-ABS-KEY(vaccin* OR immuniz* OR immunis* OR bacterin*) AND TITLE-ABS-KEY(proteom* OR immunoproteom* OR surfaceom* OR secretom* OR "mass spectrometry*" OR "LC-MS/MS" OR iTRAQ OR TMT OR DIA OR "data-independent acquisition")	[n =20 ]
S2	Scopus	Antigen-discovery supplement	TITLE-ABS-KEY(fish OR fishes OR teleost* OR aquaculture*) AND TITLE-ABS-KEY(proteome* OR immunoproteom* OR surfaceom* OR secretom* OR "mass spectrometer*" OR "LC-MS/MS" OR iTRAQ OR TMT OR DIA OR "data-independent acquisition") AND TITLE-ABS-KEY(antigen* OR immunogen* OR "vaccine candidate" OR "vaccine candidates")	[n = 20]
S3	Scopus	Biomarker/outcome supplement	TITLE-ABS-KEY(fish OR fishes OR teleost* OR aquacultur*) AND TITLE-ABS-KEY(vaccin* OR immuniz* OR immunis* OR bacterin*) AND TITLE-ABS-KEY(proteom* OR immunoproteom* OR surfaceom* OR secretom* OR "mass spectrometer*" OR "LC-MS/MS" OR iTRAQ OR TMT OR DIA OR "data-independent acquisition") AND TITLE-ABS-KEY(biomarker* OR signatur* OR predict* OR "correlate of protection" OR "correlates of protection" OR survival OR challenge)	[n = 20]
W1	Web of Science	Core vaccination-proteomics search	TS=(fish OR fishes OR teleost* OR aquacultur*) AND TS=(vaccin* OR immuniz* OR immunis* OR bacterin*) AND TS=(proteom* OR immunoproteom* OR surfaceom* OR secretom* OR "mass spectrometer*" OR "LC-MS/MS" OR iTRAQ OR TMT OR DIA OR "data-independent acquisition")	[n =49 ]
W2	Web of Science	Antigen-discovery supplement	TS=(fish OR fishes OR teleost* OR aquacultur*) AND TS=(proteom* OR immunoproteom* OR surfaceom* OR secretom* OR "mass spectrometer*" OR "LC-MS/MS" OR iTRAQ OR TMT OR DIA OR "data-independent acquisition") AND TS=(antigen* OR immunogen* OR "vaccine candidate" OR "vaccine candidates")	[n =50 ]
W3	Web of Science	Biomarker/outcome supplement	TS=(fish OR fishes OR teleost* OR aquacultur*) AND TS=(vaccin* OR immuniz* OR immunis* OR bacterin*) AND TS=(proteom* OR immunoproteomic* OR surfaceom* OR secretom* OR "mass spectrometer*" OR "LC-MS/MS" OR iTRAQ OR TMT OR DIA OR "data-independent acquisition") AND TS=(biomarker* OR signature* OR predict* OR "correlate of protection" OR "correlates of protection" OR survival OR challenge)	[n = 50]

We included peer-reviewed primary studies using proteomic or immunoproteomic approaches to identify pathogen antigens relevant to teleost vaccination or characterize vaccine-induced host protein responses. Eligible studies covered candidate antigen identification, comparisons between vaccine formulations or between vaccinated and control groups, and analyses linking protein-level measurements to vaccine-efficacy outcomes. Protective challenge trials and independent validation were not required for inclusion. Instead, their presence or absence informed the classification of evidence strength. Studies examining infection, immune stimulation or host–pathogen interactions without vaccination were considered separately as contextual evidence where they helped explain biological findings or methodological constraints. Studies lacking protein-level experimental data were not considered primary proteomic evidence. Review articles and studies in mammals or humans informed methodological and conceptual discussion but were not treated as direct evidence of vaccine-induced proteomic responses or validated correlates of protection in teleosts.

Records retrieved from the database searches were imported into Rayyan (Rayyan Systems Inc., Cambridge, MA, USA) (22). Rayyan’s duplicate-detection function was used to identify potentially duplicate records, which were verified before removal. The deduplicated records were screened by title and abstract against the predefined eligibility criteria by one reviewer (SR). Publications considered potentially relevant were subsequently assessed in full text by the same reviewer. Uncertain eligibility decisions were discussed with a second author (TP) until consensus was reached. Final decisions regarding study inclusion and evidence classification were made manually, with study characteristics and evidence classifications summarized in **Supplementary Tables S1**, **S2**. Reference lists of relevant primary studies and review articles were examined iteratively to identify additional publications not retrieved through the database searches. Because the available studies differed substantially in fish species, pathogen, vaccine platform, administration route, tissue, sampling time, proteomic workflow and protection endpoint, quantitative meta-analysis was not considered appropriate. The present study was hence considered a critical narrative synthesis rather than an exhaustive systematic review; consequently, a PRISMA flow diagram and formal risk-of-bias assessment were not applied. Findings were therefore synthesized narratively according to proteomic application, biological pathway and stage of biomarker development. Evidence was interpreted using a hierarchy progressing from vaccine-responsive proteins to immune-activation markers, protection-associated candidates, predictive correlates and mechanistic correlates. Particular attention was given to whether proteins were measured before or after pathogen challenge, linked to individual protection outcomes, analytically verified and evaluated in independent biological samples.

## 3 Current vaccine evaluation: why do we need better biomarkers

The primary objective of vaccination is to induce durable protective immunity rather than simply elicit measurable immune responses (23). In teleost fish, vaccine efficacy is traditionally evaluated using survival following pathogen challenge, humoral immune assays, histopathology, and transcriptional analyses (6). Although these approaches have supported the development of several commercial vaccines, they primarily measure vaccine outcomes rather than the molecular mechanisms responsible for protection (7). Consequently, they provide limited insight into why a vaccine succeeds or fails across different host species, pathogens, or production environments (7, 18, 24). Relative percent survival (RPS) remains the benchmark for evaluating vaccine efficacy because it directly measures the ultimate biological outcome of vaccination. However, RPS is an endpoint rather than a mechanistic readout. Identical RPS values may arise through fundamentally different immune pathways, while variations in pathogen strain, infection route, host age, water temperature, and husbandry practices further complicate comparisons across studies (25). Therefore, RPS is indispensable for assessing efficacy but provides little guidance for antigen selection or biomarker discovery (11, 26).

Antigen-specific antibody titres remain one of the most widely used indicators of adaptive immunity (27). Nevertheless, their predictive value for protection in teleosts is inconsistent because effective immunity often depends on coordinated innate responses, complement activation, antigen presentation, and cell-mediated immunity in addition to humoral responses (28). Furthermore, the limited availability of species-specific immunological reagents continues to constrain the broader application of antibody-based assays across aquaculture species (29, 30). Transcriptomic analyses have substantially improved our understanding of vaccine-induced immune activation by identifying pathways associated with cytokine signaling, pathogen recognition, and antiviral responses (31). However, transcript abundance is often a poor proxy for protein abundance or activity because immune function is extensively regulated by translational control, post-translational modification, protein turnover, and subcellular localization (32). Consequently, transcriptomics alone cannot fully explain the molecular mechanisms underlying protective immunity.

A major limitation of current vaccine evaluation in teleosts is the absence of validated molecular correlates of protection (33). For some human vaccines, validated immune markers or threshold values support efficacy assessment, whereas comparable predictive markers remain uncommon in teleost vaccinology (33, 34). Although antibody titres, cytokine production, complement activity, and lymphocyte proliferation have all been proposed as indicators of vaccine-induced immunity, few have demonstrated consistent predictive value across species, pathogens, and vaccine platforms. These limitations highlight the need for biomarkers that reflect the molecular mechanisms underlying protective immunity rather than merely its outcome. Such biomarkers should identify protective immune responses before pathogen challenge, distinguish effective from ineffective vaccines, and facilitate antigen selection and vaccine design (9, 20). Because proteins execute these immune functions, their direct measurement may complement conventional assays and help identify candidate correlates of protection (26). The complementary strengths and limitations of current vaccine evaluation approaches are summarized in **Table 3**. The following sections critically evaluate how proteomic technologies are being applied throughout the vaccine development pipeline, from antigen discovery to the identification of candidate correlates of protection.

**Table 3:** Complementary roles of conventional, functional and omics-based approaches in developing molecular correlates of protection for teleost vaccines.

Approach	Primary readout	Main contribution	Limitation and role in correlate development
Challenge trials	Survival, mortality kinetics and relative percentage survival (RPS)	Direct demonstration of vaccine efficacy under a defined pathogen challenge	High biological relevance, but costly and context-specific; provides the outcome against which candidate correlates must be tested.
Pathogen-burden assays	Culture, qPCR or digital PCR estimates of bacterial/viral load	Quantifies infection control and can distinguish survival from pathogen clearance	Affected by tissue and sampling time; valuable as an outcome component, but not a molecular correlate by itself.
Histology and clinical assessment	Lesion scores, tissue damage, behavior and disease severity	Captures morbidity and tissue protection that mortality alone may miss	Semi-quantitative and observer-sensitive; should be standardized and assessed blind wherever feasible.
Serology	Antigen-specific antibody titre, avidity or neutralization	Accessible measure of vaccine immunogenicity; occasionally linked to protection	Binding titres do not necessarily predict protection in teleosts; functional assays and outcome linkage are required.
Cellular and functional assays	Leukocyte phenotypes, cytokines, phagocytosis, complement or killing activity	Adds mechanistic information about innate and adaptive immune function	Assay availability and standardization vary among species; measurements become candidate correlates only after protection association.
Transcriptomics	RNA-seq or targeted gene-expression signatures	Broad pathway coverage and mature computational workflows	Transcript abundance may not reflect protein abundance, turnover or post-translational regulation; best integrated with protein-level evidence.
Discovery proteomics	Global protein abundance and pathway profiles by label-free LC–MS/MS, DIA, iTRAQ or TMT	Identifies expressed antigens, immune pathways and multivariate candidate signatures	Sensitive to sampling and batch effects; discovery findings require outcome linkage, targeted verification and independent replication.
Targeted proteomics	Quantification of prespecified peptides by PRM or SRM/MRM, ideally with stable-isotope standards	Supports reproducible verification and transfer of compact biomarker panels	Requires proteotypic peptides, analytical validation and predefined decision thresholds; strongest bridge toward deployable assays.

RPS, relative percentage survival; qPCR, quantitative polymerase chain reaction; RNA-seq, RNA sequencing; LC–MS/MS, liquid chromatography–tandem mass spectrometry; DIA, data-independent acquisition; iTRAQ, isobaric tags for relative and absolute quantitation; TMT, tandem mass tags; PRM, parallel reaction monitoring; SRM/MRM, selected/multiple reaction monitoring.

## 4 Proteomics as a functional platform for fish vaccinology

### 4.1 Evolution of proteomic technologies

The development of high-resolution liquid chromatography–tandem mass spectrometry has substantially expanded the scope of fish proteomics. Label-free quantification, isobaric tagging approaches such as isobaric tag for relative and absolute quantitation (iTRAQ) and tandem mass tag (TMT), and data-independent acquisition provide complementary strategies for comparative protein quantification across complex samples, although achievable proteome depth depends on sample type, fractionation, instrument performance and data-analysis workflow (35, 36). These technologies have enabled increasingly comprehensive analyses of host responses to infection, environmental stress, nutrition and vaccination in diverse aquatic species (14, 37, 38). Proteomics has thus moved beyond exploratory protein catalogs to quantify regulated pathways and candidate biomarkers. For vaccinology, this transition is particularly important because reproducible protein quantification is a prerequisite for distinguishing transient responses to immunization from molecular signatures associated with durable protection.

DIA analysis tools such as DIA-NN use neural networks and interference correction to support sensitive, reproducible protein quantification (39). Deep-learning approaches such as Prosit predict peptide fragmentation spectra and retention times, reducing reliance on experimentally generated spectral libraries (40). In teleost applications, these capabilities should be paired with species-appropriate sequence databases, explicit false-discovery controls and transparent protein-group assignments. Predicted spectra do not resolve ambiguity between paralogues that share identical peptide sequences.

### 4.2 Proteomics as a functional tool

The value of proteomics in vaccinology extends beyond protein identification because it enables direct characterization of the molecular processes engaged by vaccination (13, 36). Proteomic profiling can quantify changes in pathogen-recognition pathways, complement activation, antigen processing and presentation, intracellular signaling, cytoskeletal remodeling, metabolism, and immune effector functions (41, 42). These measurements provide a functional layer of information that cannot be inferred reliably from transcript abundance alone (36, 43, 44). Proteomics contributes to multiple stages of the vaccine-development pipeline (13). Comparative analysis of pathogen surface and secreted proteins can identify antigens that are accessible to host immunity or involved in virulence (45, 46). Immunoproteomic approaches further prioritize candidates by identifying pathogen proteins recognized by antibodies from infected or vaccinated fish. Following immunization, quantitative host proteomics can determine which immune pathways are modulated, how these responses evolve over time and whether particular signatures are associated with protection or vaccine failure (37, 47, 48).

Its main value lies in linking antigen exposure to functional host responses, rather than expanding candidate lists (49). However, this advantage is realized only when protein changes are interpreted in relation to biologically meaningful outcomes such as survival, pathogen burden, lesion severity, or durable immune memory (50). Without such outcome-based analysis, proteomic studies remain descriptive and cannot establish correlates of protection. Proteomics is also inherently complementary to other molecular approaches. Integration with transcriptomics can distinguish transcriptional regulation from changes in protein abundance, while metabolomics and lipidomics can reveal the energetic and membrane-associated adaptations accompanying immune activation (51). Combined with immunophenotyping and pathogen-load measurements, these datasets provide the foundation for systems vaccinology (52, 53), in which vaccine responses are analyzed as coordinated biological networks rather than isolated molecular changes.

### 4.3 Current limitations and research opportunities

Despite substantial technological progress, proteomics remains underused in fish vaccinology relative to mammalian vaccinology and other areas of fish biology (26, 54). Cross-study comparison is restricted by heterogeneity in fish species, pathogens, vaccine platforms, sampled tissues, analytical workflows and protein annotation (54–56). Small cohorts and isolated sampling points further limit the ability to distinguish transient inflammatory responses from molecular changes associated with durable protection (36). These experimental and analytical constraints are examined systematically in Section 8.

Human vaccinology increasingly applies systems-biology and vaccinomics frameworks that integrate host genetics, immune phenotypes, multi-omics data, and computational modeling (57, 58). A corresponding framework for teleosts should incorporate host and pathogen diversity, vaccine platform, environmental conditions and molecular readouts (59, 60). Within this framework, proteomics should function as one experimentally connected layer rather than as a stand-alone endpoint (36, 61). Section 6 therefore evaluates the evidence required to progress from vaccine-responsive proteins to protection-associated and predictive biomarkers.

## 5 Proteomics-guided antigen discovery

### 5.1 From empirical vaccine development to rational antigen discovery

Reverse vaccinology uses genomic information to predict vaccine candidates and has become increasingly accessible with the expanding availability of pathogen genome sequences (62). This approach has enabled the identification of numerous candidate antigens without the need for pathogen cultivation, greatly expanding the repertoire of potential vaccine targets (63). However, genomic predictions alone cannot determine whether candidate proteins are expressed during infection, exposed to the host immune system, or capable of eliciting protective immune responses. Consequently, many computationally predicted candidates fail during experimental validation because genomic information alone cannot establish biological relevance.

Rather than replacing genomics, proteomics provides a complementary approach by identifying proteins that are actively expressed under biologically relevant conditions. Proteomic analyses identify proteins expressed during infection or host interaction, rather than merely predicting theoretical vaccine candidates, thereby prioritizing antigens most likely involved in virulence and immune recognition (64, 65). Evidence that a candidate is expressed under infection-relevant conditions provides a stronger basis for experimental testing than genomic prediction alone (66, 67). Representative studies are organized according to the strength of candidate-specific evidence in **Table 4**, whereas the complete study-level extraction, including proteomic workflows, validation designs, limitations and bibliographic details, is provided in **Supplementary Table S1**.

**Table 4:** Evidence tiers for proteomics-guided antigen prioritization in teleost fish, illustrated by representative studies.

Evidence tier	What is demonstrated	Representative examples	What remains unproven
Immunoreactivity or antigenicity	A pathogen protein is detected by immune serum or prioritized by comparative proteomics	(69, 70)	Candidate-specific protection
Formulation-level protection	A proteomically informed antigen mixture or formulation protects fish	(68)	Contribution of an individual MS-identified protein
Candidate-specific protection	A defined recombinant antigen provides protection in a controlled challenge	(48, 72, 74, 75)	Independent replication and production-setting transferability
Heterologous or cross-protection	A defined antigen protects against more than one pathogen or strain	(71, 73)	Robustness across cohorts, routes and laboratories
Refinement or negative candidate testing	Follow-up testing improves an antigen design or shows that immunoreactivity does not predict protection	(76, 77)	Generalizability and reasons for protection or failure

These evidence tiers assess antigen candidacy, not host correlates of protection. A protective antigen is a vaccine component, whereas a correlate of protection is a measurable host attribute associated with or predictive of protection. Complete study-level information is provided in **Supplementary Table S1**.

### 5.2 Proteomic strategies for antigen discovery

One of the major contributions of proteomics to vaccine development is the identification of protein targets that directly interact with the host immune system. Pathogen surface proteins mediate host attachment, colonization, invasion, and immune evasion, making them attractive candidates for vaccine development (78). Surfaceome analysis combines biochemical enrichment with mass spectrometry to identify proteins exposed on the pathogen surface, thereby prioritizing antigens that are readily accessible to host immune recognition.

Secreted proteins represent another important class of candidate vaccine antigens. The secretome comprises proteins released into the extracellular environment, including toxins, adhesins, hydrolases, and immunomodulatory molecules (79). Many of these proteins contribute to pathogen virulence while remaining readily accessible to host immune surveillance. Consequently, comparative secretomic analyses performed under biologically relevant conditions can prioritize candidate vaccine antigens. Comparisons between virulent and avirulent strains, or pathogens grown under different environmental conditions, have identified proteins associated with host adaptation and pathogenesis (64, 80). This approach prioritizes biologically relevant antigens over merely abundant proteins, thereby increasing the likelihood of identifying protective vaccine candidates.

Another important application is immunoproteomics, which combines proteomic analysis with host immune recognition to identify immunogenic antigens. Pathogen proteins are screened by immunoblotting or immunoprecipitation followed by mass spectrometry to identify antigens recognized by antibodies from infected or vaccinated fish. Because these proteins have already elicited host immune recognition, immunoproteomics provides functional evidence of their immunogenicity and strengthens their prioritization as vaccine candidates.

### 5.3 Integrating proteomics with reverse vaccinology

Proteomics does not replace reverse vaccinology but complements it by improving the prioritization of candidate vaccine antigens (81). Reverse vaccinology rapidly screens pathogen genomes to identify proteins with characteristics associated with vaccine potential, including secretion signals, membrane localization, or predicted antigenicity (66, 82). Proteomics then determines which of these predicted candidates are actually expressed during infection and evaluates their biological relevance through protein expression and host–pathogen interactions (81).

The integration of genome-based prediction with experimental proteomic validation has substantially improved antigen discovery in both human and veterinary vaccinology (46, 48). Few teleost vaccine studies have combined genome-based prediction with experimental proteomic validation; wider use of this sequence may improve antigen prioritization for bacterial, viral and parasitic targets (83). Integrating quantitative proteomics into reverse vaccinology enables the prioritization of surface-exposed and immunologically relevant antigens while eliminating candidates that are unlikely to contribute to protective immunity.

### 5.4 Current challenges and future directions

Proteomic antigen discovery is constrained by the context-dependent expression of pathogen proteins. Proteomes can vary among strains and phenotypic states, while phase-variable regulation may alter antigen expression or exposure (84, 85). Consequently, proteins detected under laboratory conditions may not be expressed or accessible during infection. Moreover, mass-spectrometric detection or high abundance alone does not establish conservation, surface accessibility, immune recognition or protective efficacy (81, 86). Proteomic hits should therefore be treated as candidates rather than confirmed vaccine antigens.

Future discovery pipelines should combine comparative proteomics, surfaceome and secretome profiling, immunoproteomics, reverse vaccinology and structural or immunoinformatic analyses (81, 82, 87). Prioritized candidates should then pass sequential evidence gates: confirmation under infection-relevant conditions, verification of host accessibility or immune recognition, and *in vivo* vaccination–challenge testing. Proteomics strengthens the biological rationale for antigen selection but cannot independently demonstrate protective efficacy.

## 6 Proteomic biomarkers and correlates of protection: bridging immune responses and vaccine efficacy

### 6.1 What are correlates of protection?

Correlates of protection (CoPs) are measurable biological attributes statistically associated with protection, whereas mechanistic correlates have additional evidence supporting a causal contribution to protective immunity (88). For some human vaccines, validated antibody thresholds or functional immune measurements support candidate evaluation and regulatory decisions (33, 89). Teleost vaccine efficacy, by contrast, is still established primarily through pathogen-challenge experiments (26, 90, 91). The evidence synthesized in this review indicates that no pre-challenge proteomic signature has yet been independently validated as a broadly transferable predictor of protection in teleost fish.

This qualification is essential because a vaccination-induced protein change is not, by itself, evidence of protection. A protein may reflect vaccine exposure, inflammation or immune activation without being associated with subsequent survival, pathogen control or tissue preservation. Accordingly, this review distinguishes five levels of evidence:

- Vaccine-responsive protein: abundance changes following vaccination.
- Immune-activation marker: the change is linked to activation of a defined immune process.
- Protection-associated candidate: abundance differs between fish with contrasting challenge outcomes.
- Predictive correlate: a pre-challenge measurement predicts subsequent protection and retains performance in an independent cohort.
- echanistic correlate: functional evidence demonstrates that the protein or pathway contributes causally to protection.

Differential abundance supports only the first level; progression through the hierarchy requires increasingly stringent outcome linkage, temporal evidence, analytical verification and independent validation. Protection may also arise from coordinated innate, humoral, cellular, metabolic and tissue-protective responses rather than from a single protein (92–94).

Useful predictors may therefore consist of multicomponent or pathway-level signatures, although their transferability across species, vaccines and production conditions remains unproven. Section 6.2 evaluates the reported vaccine-responsive profiles, whereas Section 6.3 examines why these findings have not yet progressed to validated proteomic correlates of protection.

### 6.2 Emerging proteomic signatures associated with vaccine-induced immunity

Direct host-response proteomics following vaccination in teleosts remains sparse and methodologically heterogeneous. Longitudinal plasma profiling after immunization with a model antigen identified changes in complement, acute-phase and lipoprotein-associated proteins (37). An integrated RNA-seq and iTRAQ study of intestines from grass carp immunized with an oral double-targeted DNA (deoxyribonucleic acid) vaccine against *Vibrio mimicus* identified 1,173 differentially expressed proteins, with immune-related changes involving phagosome, antigen processing and presentation, complement and coagulation, NOD-like Receptor (NLR) and Mitogen-Activated Protein Kinase (MAPK) pathways; selected proteins were subsequently confirmed by MRM (95). DNA-vaccine-related *in vivo* and ex vivo erythrocyte studies reported antigen-processing, interferon-associated and redox responses (96, 97). Serum proteomics following nodavirus vaccination identified antioxidant and coagulation-related changes (54), while mucus and plasma studies described annexin, hemostasis and tissue-repair-associated responses following vaccination and pathogen challenge (98, 99). Functional analysis of vaccine-induced peritoneal exosomes in olive flounder further implicated major histocompatibility complex class II (MHC-II) and cluster of differentiation 80 and cluster of differentiation 86 (CD80/86) in cluster of differentiation 4 positive (CD4^+^) T lymphocyte(T-cell) activation (100).

These studies demonstrate the feasibility of proteomic profiling but do not provide equivalent levels of evidence. To be precise, Bakke et al. (37) used a model antigen without a protective pathogen challenge; the Puente-Marín studies included an ex vivo erythrocyte model (96, 97); Chérif et al. (54) compared separate vaccinated and unvaccinated cohorts without linking pre-challenge profiles to individual outcomes; and the Hoare and Carvalho datasets included post-challenge responses (98, 99). The exosome study provides stronger functional evidence but does not establish a transferable pre-challenge biomarker. Li et al. (95) confirmed selected differentially expressed proteins by Multiple reaction monitoring (MRM), thereby strengthening confidence in their measurement, but the vaccinated-versus-control design did not test whether pre-challenge protein profiles predicted individual protection or transferred to an independent cohort. No reported candidate signature has yet been prospectively replicated in an independent teleost vaccination cohort. These findings show limited pathway-level convergence among study-specific vaccine responses rather than validated or transferable correlates of protection (**Supplementary Table S2**).

#### 6.2.1 Complement and other innate immune proteins

Among innate immune proteins, complement components are biologically plausible indicators of vaccine responsiveness, but the direct proteomic evidence remains narrower than the broader fish-immunology literature suggests. The clearest evidence comes from longitudinal plasma profiling after immunization with a model antigen, in which C1q-like proteins, two C3 proteins, C6, factor B and factor H displayed distinct temporal abundance patterns (37). However, this study did not include a protective pathogen challenge. In European sea bass, complement-protein levels were not significantly altered following nodavirus vaccination (54).

Carvalho et al. (98), detected increased C1q-domain-containing protein after infection in methionine-supplemented, non-vaccinated trout, but this cannot be interpreted as a vaccine-induced complement signature. Current evidence therefore does not demonstrate consistent complement regulation across independent teleost vaccine models (98). Protein abundance must also be distinguished from functional complement activation. Increased total C3 does not establish its cleavage, deposition of C3-derived fragments on a pathogen or enhanced complement-mediated killing (101, 102). Similarly, increased factor H or factor I may indicate stronger inhibition rather than greater pathway activation (103). Functional activation is more directly demonstrated through cleavage products, convertase activity, hemolytic activity, opsonization or complement deposition (104, 105). Increased abundance may reflect acute-phase synthesis, whereas reduced abundance may result from consumption during activation. Direction of change alone is therefore insufficient to classify a complement response as protective.

Interpretation is further complicated by the duplication and functional diversification of teleost complement genes (106–108). Closely related paralogues may share peptides but differ in regulation or biological function, preventing confident assignment of some proteomic signals. Complement should therefore be regarded as a biologically credible vaccine-responsive pathway rather than a validated correlate of protection. Establishing predictive value will require paralogue-aware measurements obtained before challenge, functional complement assays and direct comparison of protected and susceptible fish in independent vaccination cohorts.

#### 6.2.2 Antigen processing, presentation and adaptive immune readouts

Evidence that vaccination modifies antigen-processing and presentation pathways in teleosts remains limited and uneven. In an ex vivo rainbow trout erythrocyte model, expression of the viral hemorrhagic septicaemia virus glycoprotein G altered proteins associated with proteasomal processing and exogenous-antigen presentation, while MHC-I-, MHC-II- and trafficking-related genes were upregulated (97). A related *in vivo* study confirmed that erythrocytes respond molecularly to DNA vaccination (96). However, these changes demonstrate engagement of pathway components rather than the formation of specific peptide–MHC complexes or subsequent antigen-specific T-cell activation. Stronger functional evidence has recently emerged from olive flounder. Exosomes produced by peritoneal cells following vaccination contained increased MHC-II and CD80/86 and promoted CD4^+^ T-cell activation; their use as an adjuvant also enhanced antibody production and vaccine efficacy (100). This study moves beyond protein-abundance associations by connecting proteomic observations with a functional immune outcome. Nevertheless, it represents a single species–vaccine model and does not establish a transferable pre-challenge correlate of protection.

Proteomic detection of immunoglobulins also requires careful interpretation. In rainbow trout immunized with a model antigen, total Immunoglobulin M (IgM) measured by LC–MS correlated with antigen-specific IgM determined by enzyme-linked immunosorbent assay (ELISA), although individual response kinetics varied and protective challenge was not evaluated (37). Total immunoglobulin abundance may therefore indicate humoral immune engagement, but it cannot independently establish antigen specificity, antibody affinity, neutralization, agglutination, opsonization or protection. Interpretation must also consider vaccination route and tissue because systemic IgM and mucosal Immunoglobulin T (IgT)/Immunoglobulin Z (IgZ) may contribute differently to protection (109, 110). Accordingly, antigen-processing proteins, MHC-associated molecules and immunoglobulin chains should currently be regarded as vaccine-responsive immune readouts rather than validated correlates of protection. Stronger validation will require direct immunopeptidomic or peptide–MHC evidence, antigen-specific T-cell measurements, antibody-function assays and prospective linkage of pre-challenge molecular profiles to individual protection outcomes.

#### 6.2.3 Metabolic and cellular-state signatures

The vaccination studies summarized above identified changes in glycolytic and pentose-phosphate-pathway enzymes, antioxidant proteins and other cellular-stress components (37, 54, 97). These findings suggest that vaccination can alter the metabolic and redox state of responding cells. Their biological interpretation, however, remains ambiguous. Increased abundance of a metabolic enzyme does not necessarily demonstrate increased pathway activity, while antioxidant proteins may indicate immune activation, oxidative stress, tissue damage or restoration of cellular homeostasis.

Metabolic and redox proteins consequently have limited specificity as stand-alone biomarkers. Their greater value may be as contextual components of multivariate signatures that combine cellular state with more immune-specific measurements, such as complement, antigen-presentation and adaptive-response proteins. Integration with metabolomics, cell-specific analyses and functional measurements would help determine whether these changes contribute to pathogen clearance or instead promote disease tolerance by limiting cellular and tissue damage.

#### 6.2.4 From individual proteins to multivariate signatures

The evidence reviewed above argues against treating vaccine-induced protection as a single-protein phenotype (26). Complement regulation, antigen processing, adaptive immunity and metabolic homeostasis represent biologically distinct dimensions of the response, none of which is likely to capture protection independently. We therefore propose that candidate signatures should comprise a small, mechanistically structured set of non-redundant proteins representing complementary immune modules rather than simply combining large numbers of differentially abundant proteins. The direction and timing of each component must also be preserved because an effector and an inhibitor within the same pathway may have opposite biological implications, while early innate and later adaptive responses represent different phases of vaccine-induced immunity.

Human systems-vaccinology studies provide proof of principle that coordinated early molecular modules can predict later vaccine responses, although this evidence has largely been derived from transcriptomic rather than proteomic measurements (111, 112). An equivalent outcome-linked multivariate proteomic signature has not yet been demonstrated in teleost vaccination. Moreover, teleost gene duplication, paralogue-specific regulation and tissue-dependent immunity suggest that initial panels will probably need to be species-, vaccine- and sampling-context-specific rather than universally transferable. The immediate goal should therefore be the development of parsimonious and biologically interpretable panels. Section 6.3 defines the evidence required to determine whether such panels have predictive value.

### 6.3 Why proteomic correlates of protection remain unvalidated

Despite more than two decades of fish vaccine-related proteomics, no proteomic correlate of protection has been independently validated in teleosts (26, 48). Most available studies remain exploratory and compare average protein abundance between vaccinated and control groups (37, 54). Such comparisons identify vaccine-responsive proteins but cannot determine whether a pre-challenge protein profile predicts protection. Testing a candidate correlate instead requires comparison within the vaccinated population: proteins must be measured before challenge and related to the subsequent outcomes of individual protected and susceptible fish. The available studies also commonly combine high-dimensional proteomic measurements with small cohorts, limited sampling points and no independent validation. Post-challenge sampling introduces an additional problem because the detected proteins may reflect infection severity, pathogen burden or tissue damage rather than the mechanisms that preceded protection (98, 99).

Comparisons between effective and ineffective formulations or between protected and susceptible fish receiving the same formulation remain particularly uncommon. Consequently, current studies provide little evidence about the predictive performance, reproducibility or transferability of candidate signatures. The major bottleneck is therefore no longer the ability of mass spectrometry to generate candidate proteins, but the design of studies capable of testing prediction. Progress will require a shift from group-level differential-abundance studies toward longitudinal, outcome-linked investigations followed by independent validation. Section 6.4 defines this evidentiary progression, while Section 8 addresses its experimental and analytical requirements.

### 6.4 From protein candidates to predictive vaccine evaluation: a proposed framework

To operationalize the evidence hierarchy defined above, candidate proteins should pass through three distinct decision gates. First, targeted analytical verification should establish whether each candidate can be measured reliably in the intended biological matrix. PRM or SRM/MRM assays should confirm peptide identity and specificity and evaluate linearity, precision, reproducibility and matrix-dependent performance, using stable-isotope-labeled peptide standards where feasible (113, 114). Candidates intended for routine decision-making should subsequently undergo fit-for-purpose analytical validation. These procedures establish assay performance; they do not show that a protein predicts protection or contributes causally to it. Second, outcome-linked predictive evaluation should measure a predefined panel before challenge and relate it to subsequent individual survival, pathogen burden and tissue-protection outcomes. The locked panel, model and decision threshold must retain performance in an independent cohort before designation as a predictive correlate. Third, mechanistic evaluation should be pursued separately through functional perturbation, depletion, inhibition or add-back experiments only when causal claims are made. This decision-gated progression is summarized in **Figure 1**; Section 8 details the analytical and experimental requirements, and Section 10 places the stages within the iterative vaccine-development roadmap.

**Figure 1:**
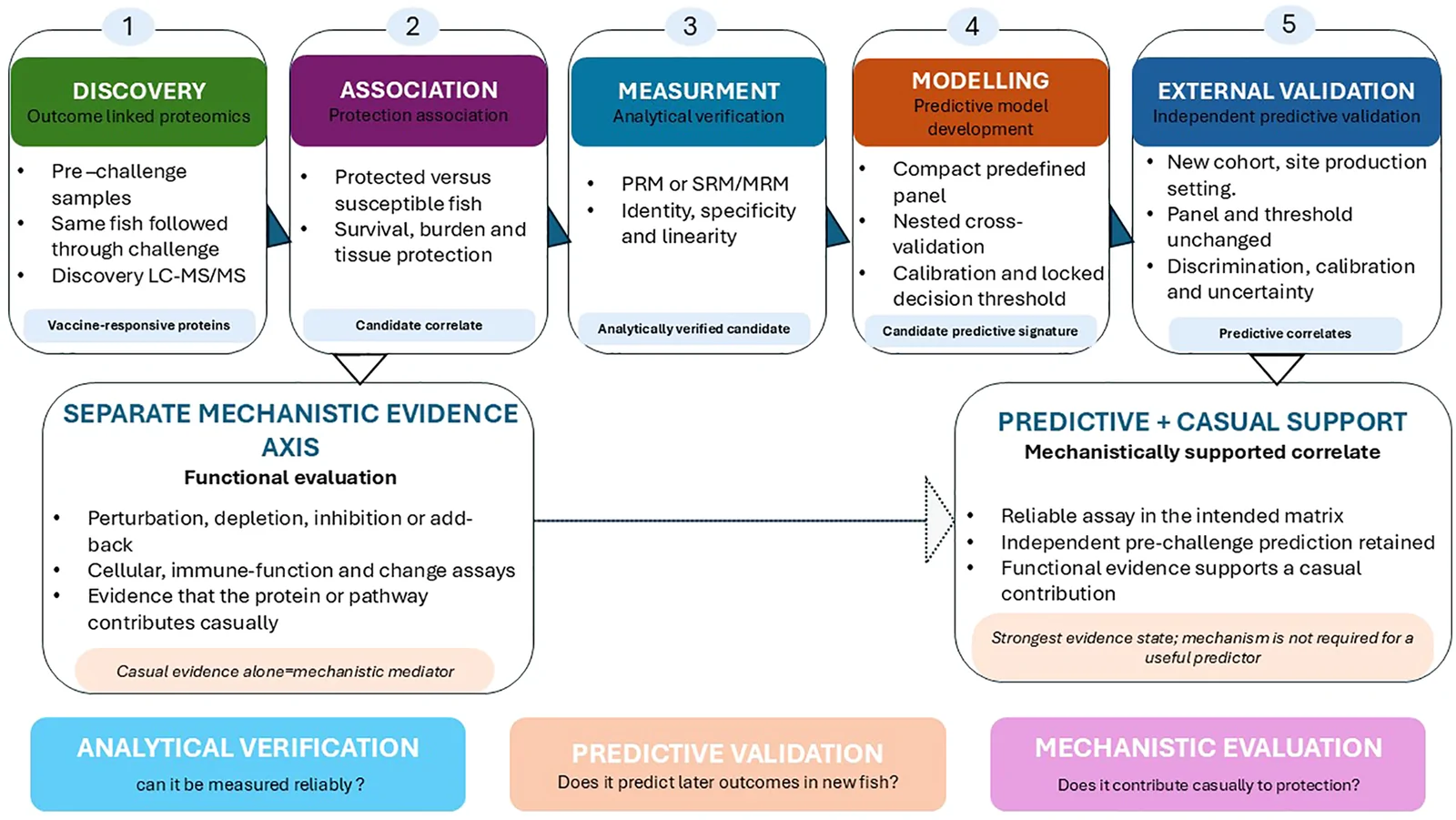
Conceptual evidence pathway for developing and validating proteomic correlates of vaccine-induced protection in teleost fish. Outcome-linked discovery proteomics first identifies vaccine-responsive proteins, after which association with protection generates candidate correlates. Targeted or orthogonal measurement establishes analytical reliability, and predictive modeling converts verified candidates into single-protein or multivariable predictive signatures. External validation then determines whether the predefined signature predicts subsequent outcomes in independent fish or cohorts; only candidates meeting this criterion are designated predictive correlates. Mechanistic evaluation constitutes a complementary evidence axis because predictive association and causal mediation address different questions. A biomarker may provide useful prediction without being a causal mediator, whereas convergence of independent predictive validation and causal evidence provides the strongest basis for biological interpretation and application.

## 7 Resistance versus tolerance signatures

Vaccine-induced protection can arise through disease resistance, in which pathogen burden is reduced, or disease tolerance, in which tissue function and health are preserved despite a given pathogen burden (115, 116). Disease tolerance in this context is distinct from immunological tolerance. Survival or RPS alone cannot distinguish these mechanisms because fish with similar survival outcomes may differ in pathogen burden and tissue damage. In rainbow trout, vaccination substantially reduced mortality following infectious hematopoietic necrosis virus exposure but had a smaller effect on viral shedding (117). This does not itself prove disease tolerance, but it demonstrates that survival and transmission-related outcomes can diverge.

Proteomics could help distinguish these mechanisms, although the proposed protein categories remain hypotheses rather than validated signatures. Resistance-associated profiles may include proteins involved in pathogen recognition, complement-mediated clearance, phagocytosis, antimicrobial defense and antigen-specific immunity. Tolerance-associated profiles may instead emphasize antioxidant defense, inflammatory regulation, metabolic homeostasis, extracellular-matrix remodeling and tissue repair. These categories are not mutually exclusive, and individual proteins can have context-dependent effects. Complement may promote pathogen clearance but also inflammatory injury, while increased antioxidant proteins may indicate either successful damage control or severe cellular stress. Protein abundance alone is therefore insufficient to classify a response as resistance or tolerance.

An informative experimental design would compare vaccinated survivors with low pathogen burdens, vaccinated survivors with high pathogen burdens and susceptible fish with severe disease, together with appropriate unvaccinated controls. Integrating longitudinal proteomics with pathogen burden and pathology would reveal whether a candidate profile precedes pathogen control, damage limitation or general disease severity. This distinction also has practical importance because a tolerance-dominated vaccine may improve survival without proportionately reducing pathogen persistence, shedding or transmission.

## 8 Current bottlenecks limiting proteomics-driven vaccine development

The failure of candidate proteins to progress toward predictive vaccine-evaluation tools results from several interconnected biological, analytical and computational barriers. These include experimental heterogeneity and limited statistical power, incomplete reference proteomes, inappropriate temporal or tissue sampling, scarce independent validation, insufficient analytical verification and poor computational reproducibility. A candidate may be lost because the assay is not reproducible, or predictive performance is not tested independently. The following subsections critically examine each barrier and propose minimum requirements for improving reproducibility and evidentiary progression in teleost vaccine proteomics.

### 8.1 Experimental heterogeneity, statistical power and reporting standards

Teleost vaccine-proteomics studies necessarily encompass diverse species, genetic backgrounds, developmental stages, vaccine platforms and pathogen models. However, this biological diversity becomes problematic when variables such as water temperature, nutrition, antigen dose, adjuvant, administration route, booster schedule and challenge protocol are unreported or confounded with treatment (11). Standardization does not require one universal protocol, but each study must control and document the variables capable of altering immune kinetics or protein abundance.

Control groups should be selected according to the biological question. Sham-treated controls can separate vaccination from handling or injection effects, whereas adjuvant-only controls distinguish antigen-specific responses from sterile inflammation. Vaccinated–unchallenged and unvaccinated–challenged groups help distinguish vaccine responses from infection-induced changes. Most importantly, protection-biomarker studies must preserve individual outcomes within the vaccinated population rather than rely exclusively on comparisons with unvaccinated controls.

The experimental unit must also be defined correctly. When vaccination or challenge is administered at the tank level, the tank not each fish within it is the independent experimental unit. Even when fish are treated individually, shared-tank effects should be represented through replicate tanks and appropriate statistical models. Pooling samples from multiple fish may increase protein coverage but removes information about individual variability and prevents direct linkage between pre-challenge profiles and subsequent outcomes. Technical replicates similarly cannot replace independent biological replication.

Proteomic datasets frequently contain hundreds or thousands of measured proteins but relatively few biological replicates. Studies should therefore justify sample size, randomize fish and samples, balance treatment groups across preparation and acquisition batches, and apply appropriate multiple-testing correction. Effect sizes and confidence intervals should accompany P values because statistical significance alone does not establish biological relevance. Randomization, replication and blocking are particularly important for preventing technical variation from being mistaken for vaccine-associated biology (118).

Reporting should follow the principles of Animal Research: Reporting of *In Vivo* Experiments (ARRIVE) 2.0 for animal experiments and Minimum Information About a Proteomics Experiment (MIAPE) for proteomic experiments (119, 120). At minimum, studies should report host and environmental characteristics, vaccination and challenge protocols, experimental-unit definition, sample handling, mass-spectrometry acquisition, reference database, protein-inference procedures, normalization, missing-data treatment and statistical analysis. **Table 5** proposes a Minimum Information for Reporting Teleost Vaccine-Proteomics (MIRT-VP) checklist, drawing on ARRIVE 2.0, MIAPE and fit-for-purpose targeted-assay principles (113, 119, 120). Core items apply to all studies, with additional requirements for targeted assays and predictive models. The checklist is a proposal for community evaluation rather than an established consensus standard.check.

**Table 5:** Proposed minimum information for reporting teleost vaccine-proteomics (MIRT-VP).

Reporting domain	Minimum information to report	Applicability
Intended use and evidence claim	Study objective, primary endpoint and whether the findings concern vaccine responsiveness, protection association, prediction or mechanism.	All studies
Host and environmental context	Species, genetic stock where known, developmental stage, size, health status, water temperature and relevant husbandry conditions.	All studies
Experimental design	Numbers of fish and tanks, experimental unit, sample-size rationale, allocation, controls, blinding, exclusions and pooling. Distinguish biological from technical replication.	All studies
Vaccination and outcome assessment	Vaccine formulation, dose, route, schedule and sampling intervals. Where challenge is performed, report the pathogen, exposure conditions, follow-up period and outcome definitions.	As applicable
Sampling and traceability	Matrix, individual or pool identifiers, collection and processing conditions, storage, freeze–thaw history and linkage between samples and outcomes	All studies
Mass-spectrometry quality control	Preparation and acquisition methods, run order, batch allocation, reference or pooled QC samples, blanks, instrument-performance checks and predefined acceptance criteria.	All studies
Identification and protein inference	Sequence-database source and version, software and search settings, false-discovery controls, peptide evidence, protein-grouping rules and treatment of shared peptides and paralogues.	All studies
Quantification and statistical analysis	Normalization, batch handling, missingness, filtering and imputation; statistical models, treatment of tank effects and repeated measures, multiple-testing correction, effect estimates and uncertainty.	All studies
Targeted-assay performance	Peptide identities, monitored ions, internal standards and when they were added; selectivity, calibration range, precision, quantification limits, stability and matrix effects. Report accuracy or recovery where assessable, with acceptance criteria and results.	Targeted-assay studies
Predictive-model development	Outcome and prediction time, candidate predictors, sample-size justification, preprocessing, feature selection, resampling, measures to prevent information leakage and the final model specification.	Predictive studies
Independent validation	Cohort independence, contextual differences tested, fixed panel and model, any updating, discrimination, calibration and confidence intervals. Report threshold-dependent performance when a decision threshold is proposed.	Predictive-validation studies
Data and analytical resources	Repository accession, raw files, peptide/protein results, abundance matrices, sample-to-file metadata, sequence databases, software versions, analysis code and model parameters where applicable.	All studies

Items should be reported according to the study’s intended use; Analytical acceptance and criteria should be prespecified and appropriate to the assay and biological matrix. Compliance with this checklist supports transparent reporting but does not itself establish predictive validity or a casual contribution to protection.

### 8.2 Incomplete reference proteomes and teleost-specific protein inference

Reference-proteome quality places an upper limit on the interpretability of fish proteomic data. Two sources of uncertainty should be distinguished. Annotation uncertainty arises when database sequences or gene models are incomplete, incorrectly named or lack functional information. Protein-inference uncertainty occurs after peptide identification when the detected peptides map to multiple related proteins. Although interconnected, these problems require different solutions (121, 122).

Teleost genomic architecture intensifies the protein-inference problem. Whole-genome duplication and subsequent lineage-specific gene expansions have generated closely related paralogues that often share peptide sequences (123, 124). Consequently, signals attributed to complement components, immunoglobulin chains, MHC-associated molecules and other expanded immune-protein families may support only a protein group rather than an individual paralogue. This distinction is biologically important because related paralogues can differ in tissue distribution, regulation and immune function. A candidate should therefore be assigned to a specific paralogue only when supported by unique peptides. When the evidence consists entirely of shared peptides, the abundance change and its interpretation should be reported at the protein-group or family level. A reproducible protein-group measurement may remain useful for prediction, provided the measured group is explicitly defined and independently validated. It should not be interpreted as evidence of a specific paralogue’s abundance or function.

At minimum, studies should report the species and genome assembly, sequence-database name and release, search date, accession identifiers, identified peptide sequences, unique or shared status of those peptides, and the rules used for protein grouping. Cross-species comparisons should also describe how orthologues were assigned because gene names alone may conceal uncertain or one-to-many relationships. For subsequent validation by PRM, SRM or MRM, species-matched proteotypic peptides that are unique to the intended protein or paralogue should be selected whenever possible.

For species with incomplete reference proteomes, species-specific transcriptomes and proteogenomic searches can refine gene models and recover missing protein sequences, as demonstrated through integrated transcriptomic and proteomic annotation of the zebrafish genome (125). However, custom databases increase the search space and may elevate false identifications unless their construction, versioning and false-discovery-rate controls are reported transparently (126). Community-curated reference proteomes and stable identifier mapping would therefore substantially improve reproducibility and cross-study synthesis. Once molecular identity has been established, the next requirement is a sampling design capable of distinguishing predictive pre-challenge signals from responses occurring after pathogen exposure.

### 8.3 Temporal resolution and longitudinal sampling

The evidentiary meaning of a protein depends on when it is measured. Baseline profiles describe the pre-existing physiological and immune state; early post-vaccination changes may reflect innate sensing, inflammation or adjuvant activity; and later pre-challenge profiles may capture adaptive maturation and response persistence. In contrast, post-challenge profiles can reflect pathogen burden, tissue damage, disease progression or recovery. Human systems-vaccinology studies have demonstrated that baseline and early molecular states can predict later vaccine responses, while longitudinal proteomics has shown that vaccine-associated protein signatures can change substantially over time (127–130).

A protein detected only after pathogen exposure may therefore provide mechanistic or prognostic information, but it cannot constitute a pre-challenge predictive correlate of protection. A comprehensive longitudinal design should include a pre-vaccination baseline, an early post-vaccination sample and a later pre-challenge measurement. Where applicable, additional samples should be collected before and after booster vaccination. Post-challenge sampling can then be used to investigate resistance, disease tolerance, vaccine failure and recovery. Universal sampling days cannot be prescribed because immune kinetics vary with fish species, developmental stage, vaccine platform and environmental conditions. Water temperature is particularly important because it influences teleost immune activity and response kinetics (131). Studies should therefore report the exact interval between vaccination, sampling, boosting and challenge together with the corresponding water temperature.

For individual-level prediction, pre-challenge measurements should be linked to each fish’s subsequent outcome. We propose prioritizing plasma for systemic profiling when fish size allows blood collection without compromising health or later outcome assessment. Longitudinal plasma proteomics has been demonstrated in immunized salmonids, although prediction of subsequent protection was not evaluated (37). Skin mucus offers a complementary matrix for mucosal vaccination studies; collection procedures should be standardized to limit variation in dilution and contamination (99). Neither matrix can currently be ranked as universally superior for predicting protection. Their relative value should instead be evaluated within the intended species, vaccine, and sampling context.

Gill, head kidney and spleen are valuable for studying local or systemic immune responses. When tissue collection requires terminal sampling, the measured profiles cannot be linked to subsequent outcomes in the same fish. Such samples are better suited to parallel cohorts that support biological interpretation. Non-lethal gill sampling requires separate assessment of its effects on fish health and challenge outcomes. Peritoneal exosomes currently offer a specialized mechanistic readout rather than an established matrix for routine longitudinal prediction (100). Where resources allow only two sampling points, we propose prioritizing a pre-vaccination baseline and a later pre-challenge sample. An early post-vaccination sample can additionally help characterize response kinetics. Sampling intervals should be justified based on species, vaccine platform, and temperature regime, rather than prescribed as universal calendar days (131).

Longitudinal analyses should preserve individual fish identity and evaluate whether baseline or pre-challenge molecular trajectories predict survival, pathogen burden and tissue pathology. Sampling volume, repeated handling and associated stress should also be controlled because they may themselves alter circulating protein profiles. Similarly, a response observed after boosting should not be described as immune memory without evidence of an accelerated or enhanced antigen-specific recall response or improved durability of protection. Longitudinal sampling can establish temporal precedence, but independent validation remains necessary before a candidate can be considered a transferable correlate of protection.

### 8.4 Reproducibility and independent validation

Reproducibility is not a single stage of biomarker development. Technical repeatability determines whether repeated preparation or measurement of the same material produces comparable results. Biological replication examines whether a finding is consistent across independent fish or experimental units. Analytical verification confirms that a selected candidate can be measured reliably using a fit-for-purpose assay. External validation provides the strongest test by evaluating a predefined candidate or model in a cohort that was not involved in discovery or assay optimization (132, 133). Repeated injections of the same sample or reanalysis of the discovery dataset cannot substitute for biological or external validation.

Defining the experimental unit is particularly important in aquaculture studies. When vaccination, pathogen exposure or environmental treatment is applied at the tank level, fish within the same tank may not represent fully independent experimental units. Increasing the number of fish sampled from one tank improves within-tank sampling but does not replace replication across tanks. Conversely, individual fish may constitute experimental units when interventions are independently allocated, and tank effects are appropriately controlled. Studies should therefore report the number of fish and tanks, treatment allocation, pooling strategy, exclusions and the experimental unit used in the statistical analysis (119, 134). Where feasible, fish and tanks should be assigned randomly to experimental groups, samples from different groups and time points should be distributed evenly across processing and mass-spectrometry batches, and pathology or other subjective outcomes should be assessed without knowledge of the vaccination group.

In aquaculture, an independent validation cohort consists of newly recruited fish from a separate experiment or production cohort that did not contribute to candidate selection, assay optimization or model development. Independence should be preserved at the relevant experimental-unit level, including independently replicated tanks where applicable. The biomarker panel, peptide identities, preprocessing and normalization procedures, model coefficients and decision threshold should be fixed before validation outcomes are examined. Reselecting features or retraining the model using this cohort constitutes model updating, and the updated model requires further independent validation. Testing a new cohort under comparable conditions assesses reproducibility within that setting. To assess transferability across farms, temperature regimes, genetic stocks, vaccine batches or pathogen strains, independent cohorts should represent the specific contextual difference under investigation. We propose that transferability claims explicitly identify the contextual difference tested rather than require an arbitrary minimum number of differing conditions. Predictive performance should be reported using appropriate measures of discrimination and calibration, with confidence intervals, rather than statistical significance alone.

Reappearance of a pathway in another dataset is not equivalent to validation of a biomarker. For example, repeated detection of complement or acute-phase proteins may demonstrate pathway-level convergence, but validation requires prospective testing of the same predefined protein, peptide or multivariate panel. Exact biomarker panels may remain species-specific even when the associated immune pathway is evolutionarily conserved. A candidate should therefore be described as validated only when its analytical reliability and predictive performance have been demonstrated in independent samples. This requirement makes targeted analytical verification the necessary bridge between discovery and external validation.

### 8.5 Analytical verification and assay transferability

Discovery proteomics can generate extensive lists of differentially abundant proteins, but it does not establish that those candidates can be measured reliably in subsequent cohorts. Candidate identity and quantitative performance should first be verified using targeted mass spectrometry. Parallel reaction monitoring (PRM) measures predefined peptides using high-resolution mass spectrometers, whereas selected or multiple reaction monitoring (SRM/MRM) measures predefined precursor–fragment-ion transitions, typically using triple-quadrupole instruments (114, 135). These methods verify analytical measurability; they do not by themselves demonstrate that a candidate predicts protection.

Assay development should begin by defining whether the measurement targets an individual paralogue or a protein group. Candidate peptides should be checked for uniqueness against the species-specific sequence database, including any available paralogue and isoform sequences. For paralogue-specific measurements, two unique peptides should be used where feasible. If only one unique peptide is used, this limitation should be justified, with supporting identity and interference checks reported. When only shared peptides are available, measurements should be reported at the protein-group level, with all compatible accessions listed. These group-level measurements should not automatically be interpreted as total gene-family abundance. Studies should report peptide sequences, unique-or-shared status, database version, precursor charge states, monitored fragment ions, retention times, and interference criteria. Quantitative performance should be evaluated in the intended biological matrix, and any discordant peptide measurements should be investigated before aggregating them into a protein-level value (113, 114).

Stable-isotope-labeled peptide standards can strengthen identification and quantification by providing reference retention times and known amounts of target peptides (136). However, a labeled peptide added after digestion does not control variation introduced during protein extraction or enzymatic digestion. More complete control may require standards added earlier in the workflow. Fit-for-purpose assay validation should assess selectivity, linearity, precision, accuracy, sensitivity, limits of detection and quantification, stability, recovery and matrix effects (113). In aquaculture, a ‘clinical grade’ targeted assay should therefore mean an assay evaluated against predefined performance criteria and not simply an assay performed using PRM or MRM.

Analytical transferability across laboratories requires evaluation testing beyond the laboratory in which the assay was developed. Standardized protocols, stable-isotope standards, shared reference materials and common quality-control samples can support comparisons across instruments and laboratories. Multisite studies have demonstrated that isotope-dilution MRM assays can achieve reproducible measurements when common materials and standardized procedures are used (137). For fish vaccine biomarkers, development should progress from targeted verification in the discovery matrix to independent biological validation and then interlaboratory evaluation. Conversion to an immunoassay, multiplex bead assay or another field-compatible format represents a new analytical platform and requires separate validation. Candidates should demonstrate reliable measurement in the intended matrix before predictive testing. Interlaboratory evaluation is additionally required when use across laboratories is intended.

### 8.6 Computational reproducibility, data sharing and interoperability

A proteomic dataset is not computationally reproducible merely because its raw mass-spectrometry files are publicly available. Reproduction of the reported results also requires the sequence database, search parameters, protein-inference rules, quantitative-processing steps and statistical decisions used to convert spectra into biological conclusions. Studies should therefore report the genome assembly and protein-database release, enzyme specificity, mass tolerances, fixed and variable modifications, false-discovery-rate thresholds, protein-grouping rules, normalization method, software names and versions, and statistical criteria (120). Analysis scripts, parameter files and intermediate outputs should be retained wherever possible so that each reported result can be traced back to the raw data (138).

Missing-value handling requires particular attention. A protein may be absent because its abundance is below the detection limit, because peptide identification was inconsistent or because of an analytical failure. These mechanisms should not automatically be treated as equivalent. Applying an inappropriate imputation method can create artificial abundance differences or apparent separation between experimental groups (139). Studies should report data completeness, filtering thresholds, the assumed reason for missingness and whether conclusions remain stable under alternative processing choices.

Pathway analysis also depends on computational decisions. Teleost proteins may be mapped to mammalian homologues when species-specific annotations are incomplete, potentially obscuring duplicated or functionally divergent fish proteins. Studies should report the identifiers used, orthology-mapping procedure, pathway database and background protein set. Enrichment analysis should be presented as support for biological interpretation rather than as proof that a pathway was functionally activated. Peptide-level evidence and protein-group assignments should remain available so that interpretations can be revised as teleost genome annotations improve.

Raw files, identification results, processed abundance matrices and supporting metadata should be deposited through a ProteomeXchange repository such as PRIDE, MassIVE or jPOST, and the dataset accession should be provided in the manuscript (140, 141). Metadata should connect every sample identifier to its corresponding raw file and include fish species and stock, developmental stage, tank, vaccine formulation, dose, administration route, sampling time, tissue, water temperature and challenge outcome. Structured formats such as SDRF-Proteomics can improve this sample-to-file mapping. Stable protein accessions, database versions and peptide sequences should also be reported because gene names alone are insufficient for comparing teleost datasets. An accession number without complete biological and analytical metadata provides data archiving, but not genuine interoperability. Transparent workflows and reusable datasets are essential if candidate vaccine signatures are to be reanalyzed, compared across studies or tested in combined cohorts.

### 8.7 Minimum standards and priority actions

Minimum standards should correspond to the evidentiary claim being made rather than impose one identical protocol on every fish–vaccine system. Discovery studies may generate candidate proteins, analytical-verification studies determine whether those candidates can be measured reliably, and validation studies test predefined candidates in independent cohorts (113, 132). Differential abundance alone should therefore not be presented as evidence of a validated biomarker or correlate of protection.

Three priorities should guide future teleost vaccine-proteomics studies:

- Design studies for prediction. Pre-challenge molecular measurements should be linked to subsequent survival, pathogen burden and pathology in the same fish wherever possible. Sample size, biological replication, tank structure, randomization and the experimental unit must be defined before data collection (119, 134).
- Verify molecular identity and analytical performance. Protein assignments should be supported by species-appropriate databases and unique peptides whenever possible. Shared peptides should be reported at the protein-group level. Prioritized candidates should progress to PRM or SRM/MRM verification using stable-isotope standards and matrix-specific performance criteria before biological validation.
- Test generalizability and enable reuse. Predefined proteins, peptides, thresholds or multivariate models should be tested in independent cohorts and subsequently across fish stocks, vaccine batches, facilities or production conditions. Raw spectra, processed matrices, analysis parameters and biological metadata should be deposited in accessible repositories according to established reporting and Findable, Accessible, Interoperable, and Reusable (FAIR) data principles (120, 140).

Collaborative and multicenter studies should prioritize validation of a small number of biologically credible panels rather than generate additional unvalidated candidate lists. In the near term, proteomic biomarkers should complement challenge experiments by improving antigen selection, formulation comparison and responder classification. Replacing or reducing pathogen challenges would require prospective field validation, evidence that biomarker-guided decisions preserve protection and acceptance by regulatory authorities. Emerging technologies cannot compensate for weak experimental design, unresolved protein identity or absent validation. The immediate bottleneck in predictive fish vaccinology is therefore not the generation of more proteomic features, but the progression of credible candidates through analytical verification and independent outcome-linked validation.

## 9 Emerging technologies and future directions

Greater biological resolution is a priority for fish vaccine proteomics. Emerging technologies can address questions that conventional bulk abundance measurements cannot resolve. Immunopeptidomics for instance, can identify naturally processed MHC-associated peptides, extending the evidence beyond changes in antigen-processing proteins or total MHC abundance (142). We propose applying this approach in teleost vaccinology to investigate whether candidate antigens contribute to the presented peptide repertoire. Such detection would provide evidence of antigen presentation but would not, by itself, establish T-cell recognition or protective efficacy.

Single-cell and spatial approaches can identify the cellular and anatomical origins of vaccine responses. Similarly, post-translational-modification proteomics can distinguish protein abundance from functional activation. Multi-omics integration with predictive modeling can test whether coordinated pre-challenge signatures forecast protection.

Greater analytical resolution does not necessarily produce stronger biological evidence. Applying high-dimensional technologies to small, poorly controlled cohorts may amplify batch effects, annotation uncertainty and statistical overfitting. Each technology should therefore address a defined question, such as which cells produce a candidate protein, where the response occurs, whether the protein is functionally activated, or whether the resulting signature improves prediction of vaccine outcome. Its value should ultimately be judged by reproducibility, independent validation and its ability to inform antigen selection, formulation optimization or vaccination strategy. The following subsections evaluate these opportunities together with the evidentiary requirements needed for their application in predictive fish vaccinology.

### 9.1 Toward integrated systems vaccinology

Systems vaccinology seeks to explain vaccine responses by integrating molecular, cellular and physiological measurements rather than examining each component independently (143). Human studies have demonstrated that early transcriptional, cellular and metabolic signatures can predict later antibody responses and that some signatures recur across vaccination seasons and populations (144, 145). However, prediction of antibody titre or immunogenicity should not automatically be interpreted as prediction of clinical protection. This distinction is equally important when adapting systems-vaccinology principles to fish.

A teleost systems-vaccinology study could integrate transcriptomics, proteomics, metabolomics, lipidomics, microbiome profiling and immunophenotyping, with each layer addressing a different component of the response (146). Integration is most informative when measurements are obtained from the same fish, tissue compartment or biologically matched cohort at aligned time points. Early transcriptional regulation may precede protein accumulation, whereas metabolic changes may represent later functional consequences. Disagreement between omics layers can therefore reflect biological timing rather than analytical failure.

The different datasets must also be linked to a shared outcome. Pre-challenge molecular profiles should be related to subsequent survival, pathogen burden and tissue pathology so that protection-associated signatures can be distinguished from general vaccine responsiveness. Non-lethal sampling of plasma, serum or mucus is particularly valuable because molecular measurements can be connected directly to the later outcome of the same fish. Destructive tissue sampling can reveal organ-specific mechanisms but generally requires parallel cohorts and cannot provide the same individual-level predictive linkage.

Teleost-specific variables including species, stock, developmental stage, vaccine route, tissue, water temperature and pathogen strain must be incorporated into both experimental design and computational modeling. Because multi-omics studies contain many more variables than biological samples, they are especially vulnerable to overfitting. Candidate networks or predictive panels should therefore be reduced to measurable features and tested in independent cohorts. The field should prioritize a small number of biologically aligned omics layers collected from a well-designed, outcome linked cohort rather than combine numerous disconnected datasets under the label of systems vaccinology. These contrasts between human systems vaccinology and current teleost vaccine research, together with their implications for predictive biomarker development in aquaculture, are summarized in **Table 6** (13, 34, 57, 130, 26, 37, 98, 99);

**Table 6:** Human versus teleost vaccinology: implications for predictive biomarker development in aquaculture.

Dimension	Human systems vaccinology	Current teleost vaccine research	Priority for teleost studies
Primary endpoints and intended prediction	Often predicts immunogenicity, responder status or durability; protection endpoints depend on vaccine and trial context.	Efficacy remains centered on survival/RPS, pathogen burden and pathology; pre-challenge molecular prediction is rare.	Define whether a signature predicts survival, pathogen control, tissue protection or durability; avoid using immune activation as a proxy for protection.
Cohorts and longitudinal sampling	Prospective cohorts, repeated sampling and independent validation are increasingly incorporated in systems-vaccinology studies.	Small, heterogeneous studies frequently use one tissue/time point or group-level comparisons.	Collect standardized pre-vaccination, post-vaccination/pre-challenge and post-challenge samples linked to individual outcomes.
Molecular resources and assay transferability	Well-annotated reference proteomes, mature reagents and clinically developed targeted assays support transfer.	Genome duplication, paralogy, variable annotation and limited species-specific reagents complicate protein inference and validation.	Report proteotypic peptides, protein groups, orthology and database versions; prioritize conserved panels while retaining species-specific context.
Multi-omics integration and modeling	Integrated transcriptomic, proteomic, metabolomic and cellular datasets are used with prespecified or internally validated models.	Proteomics is often analyzed as a stand-alone descriptive layer, with limited external validation and risk of overfitting.	Use nested cross-validation, calibration, transparent feature selection and orthogonal biological confirmation before external testing.
Validation and near-term use	Targeted assays may support stratification or trial decisions after analytical and clinical validation.	No broadly validated teleost proteomic correlate of protection is currently transferable across cohorts or production settings.	First use biomarkers to optimize antigen, adjuvant, dose, route and booster timing—not to replace challenge testing.

This strategic comparison does not imply that every human-vaccine program has a validated correlate. Representative background: (13, 34, 57, 130) for human systems vaccinology; (26, 37, 98, 99) for teleost contexts.

### 9.2 Single-cell and spatial proteomics

Bulk-tissue proteomics cannot distinguish protein regulation within a cell from changes in tissue cellular composition. For example, increased abundance of a neutrophil-associated protein may reflect greater neutrophil recruitment rather than increased expression by individual neutrophils. Mass-spectrometry-based single-cell proteomics has begun to resolve protein heterogeneity among mammalian immune and progenitor cells (147, 148). However, no peer-reviewed application of true mass-spectrometry-based single-cell proteomics to teleost vaccination was identified in the literature evaluated for this review. It should therefore be presented as an emerging opportunity rather than a currently established fish-vaccine method.

Single-cell and single-nucleus transcriptomics are presently more mature in teleosts. These approaches have resolved leukocyte diversity and generated cell-type markers in fish, including detailed characterization of Atlantic salmon head-kidney populations (149, 150). Nevertheless, transcript detection does not confirm protein abundance, post-translational modification or functional activity. A practical near-term workflow would use single-cell or single-nucleus transcriptomics to identify vaccine-responsive cell populations, followed by flow cytometry, imaging, sorted-cell proteomics or targeted PRM/MRM to verify selected proteins. This staged approach is more realistic than assuming that comprehensive single-cell mass-spectrometry proteomics can already be applied routinely in teleost vaccination studies. Cells should be analyzed as nested observations within their fish of origin, with tank-level clustering accounted for where applicable. Additional cells increase sampling depth but do not replace independent biological replication.

Spatial methods address a complementary question: where does the vaccine response occur? This is particularly relevant to immersion and oral vaccination because responses in the skin, gills and intestine may not be represented accurately by systemic organs or circulating samples. Spatially resolved methods include multiplex protein imaging, imaging mass spectrometry and laser-capture microdissection followed by LC–MS/MS (151, 152). These methods provide different levels of molecular coverage and spatial resolution: antibody-based imaging is limited by reagent availability, imaging mass spectrometry may not identify every signal at the protein level, and microdissection generally measures selected cells or tissue regions rather than intact single-cell proteomes.

The strongest applications would compare vaccinated and control fish at biologically defined pre-challenge time points and determine whether specific cell populations or anatomical niches are associated with later protection. Cell dissociation, loss of fragile populations, low protein input, incomplete reference proteomes and limited fish-specific antibodies remain substantial constraints. Spatial or single-cell maps should consequently be interpreted as mechanistic evidence unless the identified features prospectively predict challenge outcomes and are validated independently. Their contribution should be judged by whether they identify the cellular source and anatomical context of protection-associated proteins not merely by the resolution or complexity of the resulting map.

### 9.3 Post-translational modification proteomics

Conventional proteomics mainly measures total protein abundance, although many immune proteins are regulated after translation. Their activity may depend on phosphorylation, glycosylation, ubiquitination, proteolytic cleavage or other post-translational modifications. A protein may therefore remain unchanged in abundance while undergoing a major functional change (36, 153). Phosphoproteomics is particularly relevant to vaccine-induced signaling because phosphorylation regulates receptor activation, kinase cascades, transcription factors and cytoskeletal rearrangement. Toll-like receptor, JAK–STAT, MAPK and antigen-receptor pathways depend on reversible phosphorylation. Temporal design is critical because these events may occur within minutes or hours after antigen recognition and disappear before changes in total protein abundance become detectable (153).

Glycoproteomics provides information on modifications affecting protein folding, stability, secretion and receptor recognition. It is relevant to immunoglobulins, complement proteins, lectins, mucins and cell-surface receptors, where altered glycosylation could influence antibody function, complement activation or mucosal protection (154). Ubiquitinomics may clarify how immune signaling is amplified or terminated through effects on protein degradation, receptor trafficking and innate pathways. However, ubiquitination should not be interpreted automatically as degradation because different linkage types can have distinct functions (155). Proteolytic processing is also important because complement components, cytokines and other mediators are often synthesized as inactive precursors. Peptides distinguishing precursor and cleaved forms may therefore provide stronger evidence of pathway activation than total protein abundance (156).

Post-translational modification analysis remains technically demanding. Modified peptides may be scarce, require enrichment and be difficult to localize confidently. Teleost paralogues and incomplete annotations further complicate assignment. Studies should report enrichment procedures, site-localization confidence, false-discovery thresholds and peptide-level evidence (157). Candidate modifications should, where possible, be confirmed using targeted mass spectrometry, modification-specific antibodies, enzyme-activity assays or pathway perturbation. Functional interpretation requires linkage to timing, cellular source and biological outcome. Pre-challenge modifications associated with later survival or pathogen control may represent predictive candidates, whereas post-challenge changes may reflect infection severity, tissue damage or recovery. Integrated with total proteomics and outcome data, post-translational modification analysis can distinguish pathway presence from functional engagement.

### 9.4 Artificial intelligence and predictive modelling

Proteomic datasets commonly contain far more candidate features than biological replicates, making machine learning attractive but particularly vulnerable to overfitting (158). In fish vaccinology, the intended outcome must be defined before modeling. A classifier that distinguishes vaccinated from unvaccinated fish identifies vaccine exposure, whereas a predictive correlate must use pre-challenge measurements to forecast subsequent survival, pathogen burden or pathology. This distinction determines whether a model contributes to vaccine evaluation or merely describes immune activation.

Model development must prevent information leakage. Data-dependent preprocessing, including imputation and scaling, should be fitted within each training fold; feature selection and parameter tuning should likewise use only the corresponding training data. Repeated samples from the same fish should remain in the same partition. Feature selection and parameter tuning should likewise use only the corresponding training data. Where fish share tank-level exposures, validation partitions should also separate tanks to assess prediction beyond the training tanks. Analytical batches should be balanced during experimental design, with performance additionally assessed across batches where feasible. Nested cross-validation separates model selection from internal performance assessment, but it does not replace independent cohort validation (159, 160).

For binary protection outcomes, report discrimination, calibration, and where a decision threshold is specified sensitivity and specificity. Precision-recall measures are useful when outcome classes are imbalanced. Confidence intervals should account for the clustered sampling structure. Sample-size justification should consider the number of independent experimental units, outcome frequency, and candidate model complexity. For small cohorts, parsimonious models with a limited number of reproducibly measured proteins are preferable to highly flexible ones.

Model interpretability is equally important, although feature importance does not establish causality. A predictive protein may participate directly in protection, act as a surrogate for another immune process or reflect an uncontrolled technical or biological variable. Candidate panels therefore require targeted proteomic verification, independent biological validation and, where possible, functional investigation. The literature evaluated in this review contains no externally validated pre-challenge proteomic model that predicts protection in an independent teleost vaccination cohort. Artificial intelligence should therefore be presented as an emerging analytical framework rather than an established component of fish vaccine evaluation.

### 9.5 Precision vaccinology and translational applications

In aquaculture, precision vaccinology should not imply individualized vaccination of each fish. Its realistic units are the species, stock, developmental stage and production setting. This distinction is important because vaccine benefit can vary even within a single species. In Atlantic salmon challenged with *Piscirickettsia salmonis*, the protection provided by commercial vaccination differed markedly among full-sib families, ranging from no measurable benefit to an increase of approximately 13 days in survival time (161). Proteomic stratification could therefore help distinguish conserved protective pathways from stock or context dependent responses. However, apparent responder and non-responder profiles must be interpreted cautiously because temperature, nutrition, microbiota, prior pathogen exposure and husbandry may contribute alongside genetic background.

The most immediate application of such stratification is decision support. Pre-challenge proteomic profiles could help compare formulations, identify heterogeneous responses within vaccinated populations and prioritize combinations of antigen, adjuvant and administration route for challenge testing. Comparison of systemic and mucosal compartments may also indicate whether injection, immersion or oral vaccination induces responses at tissues relevant to pathogen entry (90). Longitudinal profiles could similarly inform dose and booster optimization by determining whether protection-associated responses persist or decline. These applications should nevertheless be regarded as candidate-prioritization tools rather than substitutes for efficacy testing. In the literature reviewed here, no proteomic panel has yet been independently field-validated to assign a fish stock or production group to a particular vaccination strategy.

Proteomics may also contribute to vaccine manufacturing by assessing antigen composition, degradation and batch consistency, particularly in bacterins and multivalent formulations. Targeted mass spectrometry has quantified individual antigens in a complex adjuvanted human combination vaccine, providing proof of principle for this analytical role, although comparable applications remain to be established for commercial fish vaccines (162). Translation will require reproducible targeted assays, accessible samples, prospectively defined decision thresholds and validation across farms and production environments. Such biomarkers may eventually reduce or refine some challenge-based testing, consistent with the Three Rs framework for fish-vaccine evaluation, but replacement of efficacy trials remains a longer-term objective (163). Precision vaccinology in aquaculture is therefore best regarded as an emerging decision framework rather than an established biomarker-guided practice.

## 10 A proposed roadmap for proteomics-enabled fish vaccinology

### 10.1 From an empirical pipeline to an evidence-guided development cycle

Conventional fish-vaccine development generally follows a linear sequence: antigen selection, formulation, vaccination and pathogen challenge. Efficacy is then determined using survival or RPS, pathogen burden and pathology (26). Challenge experiments remain indispensable for demonstrating protection, but their endpoints provide limited guidance when a formulation fails. Poor performance may result from unsuitable antigen selection, inadequate antigen presentation, inappropriate adjuvant activity, incorrect tissue targeting, insufficient response persistence or excessive inflammatory damage. Endpoint measurements alone cannot readily distinguish among these causes.

A proteomics-enabled approach introduces molecular evidence at several decision points. Pathogen-focused proteomics can strengthen antigen prioritization, while host-response profiling can assess the quality and persistence of vaccine-induced immunity before challenge. Linking these profiles to subsequent protection can then distinguish general vaccine responsiveness from candidate predictive biomarkers. The resulting evidence can guide iterative changes in antigen selection, formulation, delivery route, dose and booster strategy.

Challenge testing remains the efficacy endpoint, but proteomics can help explain unsuccessful formulations and identify which component of the vaccine design requires revision. The feedback loops incorporated into this cycle are illustrated in **Figure 2**.

**Figure 2:**
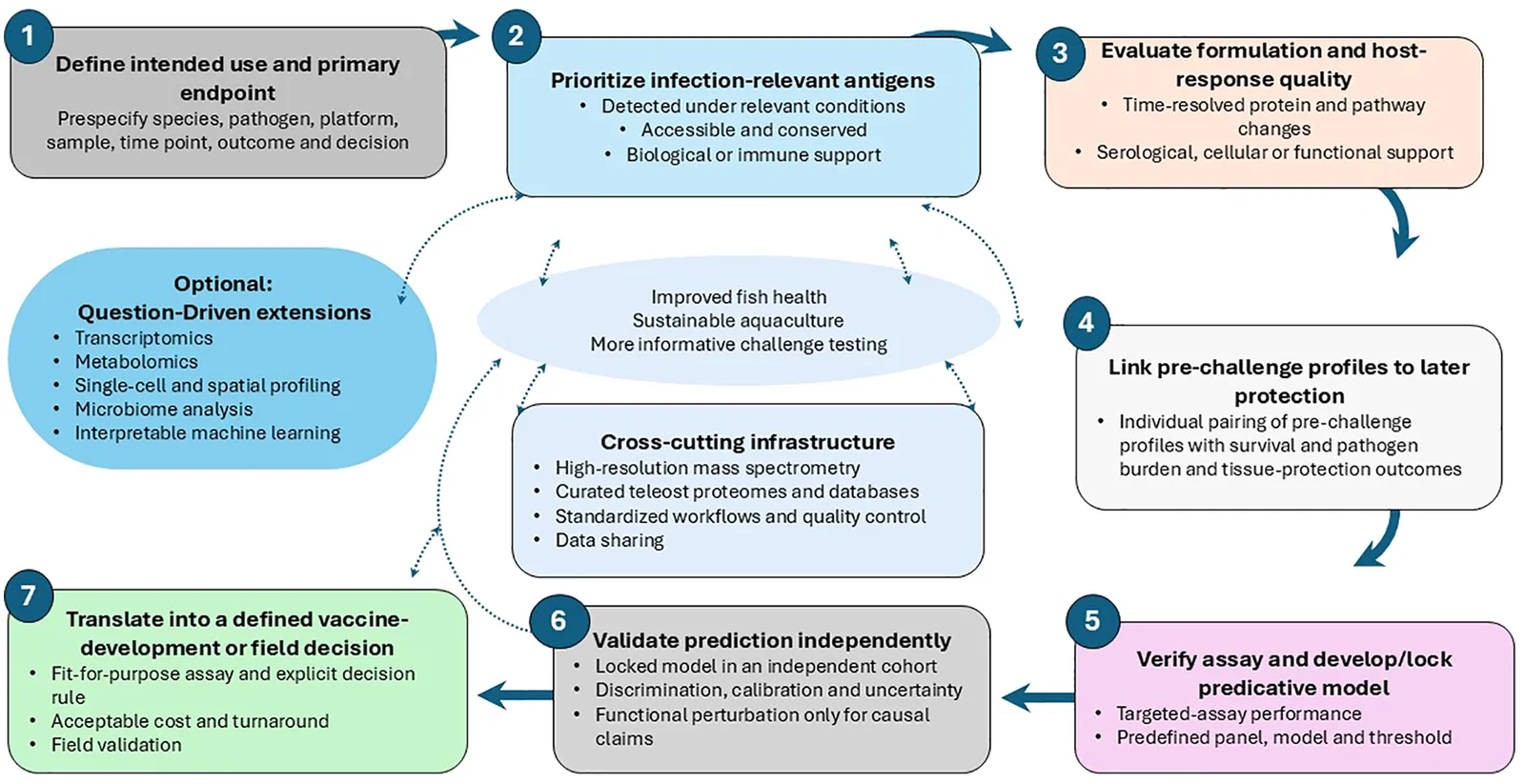
Proteomics-enabled decision-gated roadmap for fish vaccine development. The roadmap begins by defining the intended use and primary endpoint and then progresses through infection-relevant antigen prioritization, formulation and host-response assessment, outcome-linked pre-challenge profiling, analytical verification and predictive-model development, independent predictive validation, and translation into a defined vaccine-development or field decision. Multi-omics and advanced profiling are optional, question-driven extensions rather than mandatory stages. Predictive and mechanistic evidence remain separate: functional perturbation is required only when a causal mechanism is claimed. High-resolution mass spectrometry, curated teleost proteomes and databases, standardized workflows, quality control and data sharing provide enabling infrastructure across the roadmap. Failure at a decision gate redirects development to the stage requiring refinement, while validated signatures can support antigen, formulation, dose, route, booster and field decisions and help refine challenge comparisons.

### 10.2 A proposed proteomics-enabled roadmap for fish vaccine development

The proposed roadmap integrates pathogen-focused proteomics, host-response profiling and biomarker validation across seven connected stages. Progression should depend on the strength and relevance of the evidence generated rather than on the number of proteins identified.

#### 10.2.1 Stage 1: define intended use and primary endpoint

The target species, production stage, pathogen, vaccine platform, administration route and required duration of protection should be specified before proteomic analysis begins. The intended outcome must also be defined, including whether vaccination is expected to reduce pathogen burden, limit tissue damage or achieve both resistance and disease tolerance. Antigen discovery, formulation comparison and predictive-biomarker development require different experimental designs and should not be combined without a predefined objective.

#### 10.2.2 Stage 2: prioritize infection-relevant antigens

Reverse-vaccinology predictions should be strengthened using surfaceome, secretome, comparative-proteomic and immunoproteomic evidence. Candidates should progress when they are expressed under relevant conditions, accessible to immune recognition and supported by biological or immunological evidence. These characteristics justify experimental testing but do not demonstrate protective efficacy.

#### 10.2.3 Stage 3: evaluate formulation and host-response quality

Prioritized antigens should be evaluated in appropriate formulations while considering adjuvant, dose, delivery route and booster schedule. Longitudinal profiling should include early late and pre-challenge sampling to distinguish transient inflammation from coordinated and persistent immunity. Mucosal compartments should be examined when they represent the vaccination route or pathogen-entry site. Formulations dominated by stress or tissue-damage signatures should be reconsidered even when they produce strong immune activation.

#### 10.2.4 Stage 4: link pre-challenge profiles to later protection

Proteomic measurements obtained before pathogen exposure should be linked to individual survival, pathogen burden and pathology. Comparisons among protected, tolerant and susceptible fish can distinguish general vaccine responsiveness from candidate signatures associated with protection. Post-challenge profiles remain valuable for mechanistic interpretation but cannot independently establish prospective prediction.

#### 10.2.5 Stage 5: verify assay and develop/lock predictive model

Candidate proteins should undergo targeted analytical verification for peptide specificity, quantitative reproducibility and assay stability before model development. Verified candidates may then be incorporated into predefined models that prevent information leakage and account for biological clustering. A model derived from a single cohort should be described as a candidate predictive signature, not as a validated correlate of protection.

#### 10.2.6 Stage 6: validate prediction independently; evaluate mechanism separately when claimed

Candidate signatures should be tested in independent cohorts using the locked panel, model and decision threshold, with discrimination, calibration and uncertainty reported against predefined criteria. Transfer across relevant biological and production conditions should then be evaluated. Predictive and mechanistic evidence should remain distinct: a protein may predict protection without mediating it, whereas biological plausibility alone does not establish predictive value. Functional perturbation is required only when a direct protective mechanism is claimed.

#### 10.2.7 Stage 7: translate into a defined vaccine-development or field decision

Translation requires the biomarker’s intended use to be specified, such as antigen prioritization, formulation comparison, dose selection, booster timing, batch assessment or identification of poorly responding populations. The assay must be reliable, affordable and compatible with accessible, stable and preferably non-lethal samples. Multicenter field testing is then required to determine whether performance is maintained under commercial conditions.

These stages connect biological discovery to application through feedback between successive decisions. **Figure 2** illustrates this iterative roadmap, whereas **Table 7** defines the evidence requirements, decision gates and corrective actions associated with progression or failure.

**Table 7:** Decision gates for progressing proteomic candidates from discovery to practical use in teleost vaccine development.

Stage	Decision gate	Minimum evidence for progression	Action if criterion is not met
Define intended use and primary endpoint	What biological or product decision will the marker support?	Prespecified species, pathogen, vaccine platform, outcome, sample type, time point and intended decision.	Narrow the claim and define one primary endpoint before discovery.
Prioritize infection-relevant antigens	Is the candidate expressed, accessible, conserved and immunologically relevant?	Proteomic detection under infection-relevant conditions plus surface/secretory evidence, conservation and/or immune recognition.	Re-rank candidates; reject abundant but inaccessible, poorly conserved or non-immunoreactive proteins.
Evaluate formulation and host-response quality	Does vaccination produce the intended molecular and functional response?	Time-resolved protein/pathway changes supported by serological, cellular or functional assays.	Modify antigen, adjuvant, dose, route, delivery system or booster timing.
Link pre-challenge profiles to later protection	Does a pre-challenge measurement distinguish later outcomes?	Individual-level pairing of pre-challenge samples with survival, pathogen load and tissue-protection outcomes; prespecified covariates.	Treat findings as vaccine-responsive only; redesign sampling or increase outcome contrast and cohort size.
Verify assay and develop/lock predictive model	Is the panel analytically stable and statistically credible?	Targeted PRM or SRM/MRM, proteotypic peptides, stable-isotope standards where feasible, precision/linearity, nested cross-validation and calibration.	Replace unstable peptides, reduce features, address batch effects and refit without information leakage.
Validate prediction independently; evaluate mechanism separately when claimed	Does performance transfer, and is any causal claim justified?	Predefined panel and threshold in an independent cohort; discrimination, calibration and confidence intervals. Functional perturbation only for mechanistic claims.	Restrict the context of use, retain a non-causal predictor label or return to discovery.
Translate into a defined vaccine-development or field decision	Will the assay change a vaccine-development or field decision?	Robust low-volume assay, acceptable cost/turnaround, field-relevant sampling and a decision rule tied to antigen, dose, route or booster optimization.	Use the panel as a research tool until operational utility and added value over conventional measures are demonstrated.

Targeted verification should preferentially use PRM or SRM/MRM with stable-isotope-labeled peptide standards where feasible. Statistical performance should be reported with discrimination, calibration and uncertainty.

### 10.3 Decision gates and iterative implementation

The proposed roadmap is hierarchical but not strictly linear. Progression should occur only when the evidence requirement for each stage is satisfied; failure should redirect development to the stage requiring refinement. **Table 7** summarizes the principal decision gates, minimum evidence requirements and corresponding corrective actions. For example, an antigen predicted from genomic data but not detected under infection-relevant conditions should return to antigen discovery. A formulation dominated by acute-phase, stress or tissue-damage signatures should prompt reconsideration of the adjuvant, dose or delivery route. Similarly, a strong systemic response accompanied by poor protection against a mucosal pathogen may indicate inappropriate tissue targeting. Candidates that cannot be quantified reproducibly should be removed before predictive modeling, whereas failure during external validation should lead to model simplification, recalibration or restriction to a narrower biological context.

Predictive and mechanistic evidence must also be evaluated separately. A protein may predict protection without directly mediating it and may remain useful as a biomarker. Conversely, a biologically plausible protein should not progress toward translation if it cannot be measured reliably or fails to predict outcome. Negative findings are therefore informative because they identify whether failure arose from antigen selection, formulation, assay performance, predictive modeling or biological transferability. Initial implementation should focus on antigen and formulation comparison under controlled conditions, followed by independent cohort and field validation. Farm-level or regulatory application will require predefined performance thresholds, transferable assays and evidence that the biomarker provides information beyond established efficacy endpoints. These decision gates and feedback loops make the roadmap an adaptive development framework rather than a fixed sequence.

## 11 Discussion

Proteomics can connect three questions in teleost vaccine development: which pathogen proteins warrant testing, which host pathways respond to vaccination and which pre-challenge measurements are associated with protection. Pathogen-focused approaches can strengthen antigen prioritization by identifying proteins that are expressed, accessible and immunologically recognized, while host-response proteomics can reveal how vaccination affects complement regulation, antigen processing, adaptive immunity, metabolism and tissue homeostasis. However, the current evidence remains predominantly exploratory. Most studies identify proteins that differ between vaccinated and unvaccinated fish without determining whether those proteins predict subsequent protection. No pre-challenge proteomic signature identified in the literature reviewed here has yet been prospectively replicated in an independent teleost vaccination cohort. Differential abundance must therefore be treated as the beginning of biomarker discovery rather than evidence of a correlate of protection.

Candidate development is currently limited chiefly by cohort design, longitudinal outcome linkage and external validation. Small cohorts, isolated sampling points, inappropriate tissue selection and limited external validation restrict the interpretation of current findings. Future studies should use adequately powered, longitudinal designs that link individual pre-challenge profiles to survival, pathogen burden and pathology. Discovery candidates must subsequently undergo targeted analytical verification and independent biological and field validation. Single-cell, spatial and post-translational-modification proteomics, together with multi-omics integration and machine learning, can provide additional resolution, but greater technological complexity cannot compensate for weak experimental design. The roadmap proposed in this review places proteomics at defined and iterative decision points across fish-vaccine development. Its immediate value lies in strengthening antigen and formulation prioritization, distinguishing protective responses from non-protective immune activation and identifying why a vaccine fails. Proteomic biomarkers are not yet ready to replace pathogen-challenge experiments, which remain essential for demonstrating efficacy. However, validated signatures could progressively refine experimental design, reduce uninformative challenge comparisons and support decisions concerning formulation, dose, delivery route and booster timing. As a critical narrative review, this synthesis emphasizes conceptual and evidentiary interpretation rather than an exhaustive inventory; no formal risk-of-bias assessment was undertaken.

The value of proteomics in fish vaccinology will depend on whether reproducible signatures predict protection and improve vaccine-development decisions. Progress will be demonstrated when a predefined protein signature predicts protection in an independent cohort and improves antigen, formulation or dosing decisions.

## References

[1] Lafferty KD Harvell CD Conrad JM Friedman CS Kent ML Kuris AM . Infectious diseases affect marine fisheries and aquaculture economics. Ann Rev Mar Sci. (2015) 7:471–96. doi: 10.1146/annurev-marine-010814-015646 25251276

[2] Miccoli A Manni M Picchietti S Scapigliati G . State-of-the-art vaccine research for aquaculture use: The case of three economically relevant fish species. Vaccines (Basel). (2021) 9:140. doi: 10.3390/vaccines9020140 33578766 PMC7916455

[3] Reverter M Sarter S Caruso D Avarre J-C Combe M Pepey E . Aquaculture at the crossroads of global warming and antimicrobial resistance. Nat Commun. (2020) 11:1870. doi: 10.1038/s41467-020-15735-6 32312964 PMC7170852

[4] FAO . The State of World Fisheries and Aquaculture 2022. Towards Blue Transformation. Rome: FAO. (2022). 266p. doi: 10.4060/cc0461en

[5] Hoelzer K Bielke L Blake DP Cox E Cutting SM Devriendt B . Vaccines as alternatives to antibiotics for food producing animals. Part 2: New approaches and potential solutions. Vet Res. (2018) 49:70. doi: 10.1186/s13567-018-0561-7 30060759 PMC6066917

[6] Rao S Byadgi O Pulpipat T Wang P Chen S . Efficacy of a formalin‐inactivated *Lactococcus garvieae* vaccine in farmed grey mullet (*Mugil cephalus*). J Fish Dis. (2020) 43:1579–89. doi: 10.1111/jfd.13260 32935338

[7] Ma J Bruce TJ Jones EM Cain KD . A review of fish vaccine development strategies: Conventional methods and modern biotechnological approaches. Microorganisms. (2019) 7:569. doi: 10.3390/microorganisms7110569 31744151 PMC6920890

[8] Mondal H Thomas J . A review on the recent advances and application of vaccines against fish pathogens in aquaculture. Aquacult Int. (2022) 30:1971–2000. doi: 10.1007/s10499-022-00884-w 35528247 PMC9059915

[9] Dalmo RA . DNA vaccines for fish: Review and perspectives on correlates of protection. J Fish Dis. (2018) 41:1–9. doi: 10.1111/jfd.12727 29064091

[10] Rathor GS Swain B . Advancements in fish vaccination: Current innovations and future horizons in aquaculture health management. Appl Sci. (2024) 14:5672. doi: 10.3390/app1413567230654563

[11] Kyslík J Adamek M Korytář T . Decoding the black box of fish vaccines efficacy in basic and applied contexts. Rev Aquacult. (2026) 18:e70185. doi: 10.1111/raq.7018540046247

[12] Pham TH Rao S Cheng T-C Wang P-C Chen S-C . The moonlighting protein fructose 1,6-bisphosphate aldolase as a potential vaccine candidate against Photobacterium damselae subsp. piscicida in Asian sea bass (Lates calcarifer). Dev Comp Immunol. (2021) 124:104187. doi: 10.1016/j.dci.2021.104187 34186149

[13] Nakaya HI Pulendran B . Systems vaccinology: Its promise and challenge for HIV vaccine development. Curr Opin HIV AIDS. (2012) 7:24–31. doi: 10.1097/COH.0b013e32834dc37b 22156839 PMC3253345

[14] Six A Bellier B Thomas‐Vaslin V Klatzmann D . Systems biology in vaccine design. Microb Biotechnol. (2012) 5:295–304. doi: 10.1111/j.1751-7915.2011.00321.x 22189033 PMC3815789

[15] Sukumaran A Woroszchuk E Ross T Geddes-McAlister J . Proteomics of host–bacterial interactions: New insights from dual perspectives. Can J Microbiol. (2021) 67:213–25. doi: 10.1139/cjm-2020-0324 33027598

[16] Birhanu AG . Mass spectrometry-based proteomics as an emerging tool in clinical laboratories. Clin Proteom. (2023) 20:32. doi: 10.1186/s12014-023-09424-x 37633929 PMC10464495

[17] Zhang K Zhang J Wang L Liang Q Niu Y Gu L . Integrative transcriptomics and proteomics analysis reveals immune response process in bovine viral diarrhea virus-1-infected peripheral blood mononuclear cells. Veterinary Sci. (2023) 10:596. doi: 10.3390/vetsci10100596 37888548 PMC10611041

[18] Munang’andu H Galindo-Villegas J David L . Teleosts genomics: Progress and prospects in disease prevention and control. IJMS. (2018) 19:1083. doi: 10.3390/ijms19041083 29617353 PMC5979277

[19] Bøgwald J Dalmo RA . Protection of teleost fish against infectious diseases through oral administration of vaccines: Update 2021. IJMS. (2021) 22:10932. doi: 10.3390/ijms222010932 34681594 PMC8535532

[20] Golding H Khurana S Zaitseva M . What is the predictive value of animal models for vaccine efficacy in humans?: The importance of bridging studies and species-independent correlates of protection. Cold Spring Harb Perspect Biol. (2018) 10:a028902. doi: 10.1101/cshperspect.a028902 28348035 PMC5880166

[21] Agrawal I Lee AQ Gong Z . Identifying universal fish biomarker genes in response to PCB126 exposure by comparative transcriptomic analyses. CIMB. (2024) 46:7862–76. doi: 10.3390/cimb46080466 39194683 PMC11352907

[22] Ouzzani M Hammady H Fedorowicz Z Elmagarmid A . Rayyan—a web and mobile app for systematic reviews. Syst Rev. (2016) 5:210. doi: 10.1186/s13643-016-0384-4 27919275 PMC5139140

[23] Amanna IJ Slifka MK . Successful vaccines. In: Hangartner L Burton DR , editors. Vaccination Strategies Against Highly Variable Pathogens. Springer International Publishing, Cham (2018). p. 1–30. doi: 10.1007/82_2018_102

[24] Munang’andu HM Mutoloki S Evensen Ø . An overview of challenges limiting the design of protective mucosal vaccines for finfish. Front Immunol. (2015) 6. doi: 10.3389/fimmu.2015.00542 26557121 PMC4617105

[25] Nazareth SC Rao S Cheng L Wang P Chen S . *Nocardia seriolae* cell wall lipids: An effective protective mechanism in resistance and virulence. J Fish Dis. (2023) 46:405–16. doi: 10.1111/jfd.13753 36628981

[26] Munang’andu HM Evensen Ø . Correlates of protective immunity for fish vaccines. Fish Shellfish Immunol. (2019) 85:132–40. doi: 10.1016/j.fsi.2018.03.060 29621636

[27] Erfanmanesh A Beikzadeh B Khanzadeh M Alishahi M . Immuno-protective response of Asian seabass (Lates calcarifer) to inactivated vaccines against Streptococcus iniae and Vibrio harveyi. BMC Vet Res. (2024) 20:89. doi: 10.1186/s12917-024-03935-x 38459562 PMC10921715

[28] Amanna IJ Slifka MK . Contributions of humoral and cellular immunity to vaccine-induced protection in humans. Virology. (2011) 411:206–15. doi: 10.1016/j.virol.2010.12.016 21216425 PMC3238379

[29] Magnadóttir B . Innate immunity of fish (overview). Fish Shellfish Immunol. (2006) 20:137–51. doi: 10.1016/j.fsi.2004.09.006 15950491

[30] Mokhtar DM Enas AA . An overview of the structural and functional aspects of immune cells in teleosts. Histol Histopathol. (2021) 36:399–414. doi: 10.14670/HH-18-302 33415722

[31] Rao SS Lunde HS Dolan DWP Fond AK Petersen K Haugland GT . Transcriptome-wide analyses of early immune responses in lumpfish leukocytes upon stimulation with poly(I:C). Front Immunol. (2023) 14:1198211. doi: 10.3389/fimmu.2023.1198211 37388730 PMC10300353

[32] Rao SS Skinnemoen L Fond AKS Haugland GT . Analyses of the Mx family members in lumpfish: Molecular characterization, phylogeny, and gene expression analyses. Dev Comp Immunol. (2024) 159:105225. doi: 10.1016/j.dci.2024.105225 38992732

[33] King DF Groves H Weller C . The role of correlates of protection in overcoming barriers to vaccine development and demonstrating efficacy. NPJ Vaccines. (2024) 9:78. doi: 10.1038/s41541-024-00873-5 38615121 PMC11016058

[34] Van Tilbeurgh M Lemdani K Beignon A-S Chapon C Tchitchek N Cheraitia L . Predictive markers of immunogenicity and efficacy for human vaccines. Vaccines. (2021) 9:579. doi: 10.3390/vaccines9060579 34205932 PMC8226531

[35] Çağatay İT . Use of proteomic-based MALDI-TOF mass spectra for identification of bacterial pathogens in aquaculture: A review. Aquacult Int. (2024) 32:7835–71. doi: 10.1007/s10499-024-01544-x30311153

[36] Aebersold R Mann M . Mass-spectrometric exploration of proteome structure and function. Nature. (2016) 537:347–55. doi: 10.1038/nature19949 27629641

[37] Bakke FK Monte MM Stead DA Causey DR Douglas A Macqueen DJ . Plasma proteome responses in salmonid fish following immunization. Front Immunol. (2020) 11:581070. doi: 10.3389/fimmu.2020.581070 33133099 PMC7579410

[38] Das BK Kumar V Mandal B . Proteomics in fish health and aquaculture. In: Das BK Kumar V Roy S , editors. Bioinformatics Tools for Predictive Ecology and Fisheries. Springer Nature Singapore, Singapore (2026). p. 141–65. doi: 10.1007/978-981-95-2573-7_8

[39] Demichev V Messner CB Vernardis SI Lilley KS Ralser M . DIA-NN: Neural networks and interference correction enable deep proteome coverage in high throughput. Nat Methods. (2020) 17:41–4. doi: 10.1038/s41592-019-0638-x 31768060 PMC6949130

[40] Gessulat S Schmidt T Zolg DP Samaras P Schnatbaum K Zerweck J . Prosit: Proteome-wide prediction of peptide tandem mass spectra by deep learning. Nat Methods. (2019) 16:509–18. doi: 10.1038/s41592-019-0426-7 31133760

[41] Bennike TB . Advances in proteomics: Characterization of the innate immune system after birth and during inflammation. Front Immunol. (2023) 14:1254948. doi: 10.3389/fimmu.2023.1254948 37868984 PMC10587584

[42] Merle NS Church SE Fremeaux-Bacchi V Roumenina LT . Complement system part I - Molecular mechanisms of activation and regulation. Front Immunol. (2015) 6. doi: 10.3389/fimmu.2015.00262 26082779 PMC4451739

[43] Urbiola-Salvador V Miroszewska D Jabłońska A Qureshi T Chen Z . Proteomics approaches to characterize the immune responses in cancer. Biochim Biophys Acta (BBA) - Mol Cell Res. (2022) 1869:119266. doi: 10.1016/j.bbamcr.2022.119266 35390423

[44] Leprêtre M Hamar J Urias MB Kültz D . Comparative proteomics of salinity stress responses in fish and aquatic invertebrates. Proteomics. (2025) 25:185–205. doi: 10.1002/pmic.202400255 39924752 PMC12645818

[45] Pizza M Scarlato V Masignani V Giuliani MM Aricò B Comanducci M . Identification of vaccine candidates against serogroup B meningococcus by whole-genome sequencing. Science. (2000) 287:1816–20. doi: 10.1126/science.287.5459.1816 10710308

[46] Rappuoli R . Reverse vaccinology, a genome-based approach to vaccine development. Vaccine. (2001) 19:2688–91. doi: 10.1016/S0264-410X(00)00554-5 11257410

[47] Birnie‐Gauvin K Costantini D Cooke SJ Willmore WG . A comparative and evolutionary approach to oxidative stress in fish: A review. Fish Fish. (2017) 18:928–42. doi: 10.1111/faf.1221540046247

[48] Chen Z Peng B Wang S Peng X . Rapid screening of highly efficient vaccine candidates by immunoproteomics. Proteomics. (2004) 4:3203–13. doi: 10.1002/pmic.200300844 15378699

[49] Reis-de-Oliveira G Carregari VC Sousa GRDRD Martins-de-Souza D . OmicScope unravels systems-level insights from quantitative proteomics data. Nat Commun. (2024) 15:6510. doi: 10.1038/s41467-024-50875-z 39095347 PMC11297029

[50] Johnson CH Ivanisevic J Siuzdak G . Metabolomics: Beyond biomarkers and towards mechanisms. Nat Rev Mol Cell Biol. (2016) 17:451–9. doi: 10.1038/nrm.2016.25 26979502 PMC5729912

[51] Sanches PHG De Melo NC Porcari AM De Carvalho LM . Integrating molecular perspectives: Strategies for comprehensive multi-omics integrative data analysis and machine learning applications in transcriptomics, proteomics, and metabolomics. Biology. (2024) 13:848. doi: 10.3390/biology13110848 39596803 PMC11592251

[52] He Y . Omics‐based systems vaccinology for vaccine target identification. Drug Dev Res. (2012) 73:559–68. doi: 10.1002/ddr.2104942587375

[53] Jean Beltran PM Federspiel JD Sheng X Cristea IM . Proteomics and integrative omic approaches for understanding host–pathogen interactions and infectious diseases. Mol Syst Biol. (2017) 13:MSB167062. doi: 10.15252/msb.20167062 28348067 PMC5371729

[54] Chérif N Ghedira K Agrebi H Najahi S Mejri H Azouz S . Proteomic profiling of the serological response to a chemically-inactivated nodavirus vaccine in European sea bass Dicentrarchus labrax. Vet Res Commun. (2025) 49:125. doi: 10.1007/s11259-025-10688-x 40035983

[55] Chandramouli K Qian P-Y . Proteomics: Challenges, techniques and possibilities to overcome biological sample complexity. Hum Genomics Proteomics. (2009) 1:239204. doi: 10.4061/2009/239204 20948568 PMC2950283

[56] Gajahin Gamage NT Miyashita R Takahashi K Asakawa S Senevirathna JDM . Proteomic applications in aquatic environment studies. Proteomes. (2022) 10:32. doi: 10.3390/proteomes10030032 36136310 PMC9505238

[57] Pulendran B . Systems vaccinology: Probing humanity’s diverse immune systems with vaccines. Proc Natl Acad Sci USA. (2014) 111:12300–6. doi: 10.1073/pnas.1400476111 25136102 PMC4151766

[58] Montin D Santilli V Beni A Costagliola G Martire B Mastrototaro MF . Towards personalized vaccines. Front Immunol. (2024) 15:1436108. doi: 10.3389/fimmu.2024.1436108 39421749 PMC11484009

[59] Sommerset I Krossøy B Biering E Frost P . Vaccines for fish in aquaculture. Expert Rev Vaccines. (2005) 4:89–101. doi: 10.1586/14760584.4.1.89 15757476

[60] Kumar A Middha SK Menon SV Paital B Gokarn S Nelli M . Current challenges of vaccination in fish health management. Animals. (2024) 14:2692. doi: 10.3390/ani14182692 39335281 PMC11429256

[61] Gillet LC Navarro P Tate S Röst H Selevsek N Reiter L . Targeted data extraction of the MS/MS spectra generated by data-independent acquisition: A new concept for consistent and accurate proteome analysis. Mol Cell Proteomics. (2012) 11:O111.016717. doi: 10.1074/mcp.O111.016717 22261725 PMC3433915

[62] Rappuoli R . Reverse vaccinology. Curr Opin Microbiol. (2000) 3:445–50. doi: 10.1016/S1369-5274(00)00119-3 11050440

[63] Heinson AI Woelk CH Newell M-L . The promise of reverse vaccinology. Int Health. (2015) 7:85–9. doi: 10.1093/inthealth/ihv002 25733557

[64] Biron DG Nedelkov D Missé D Holzmuller P . Proteomics and host—pathogen interactions. In: Genetics and Evolution of Infectious Disease. Amsterdam: Elsevier (2011). p. 263–303. doi: 10.1016/B978-0-12-384890-1.00011-X

[65] Chen B Zhang D Wang X Ma W Deng S Zhang P . Proteomics progresses in microbial physiology and clinical antimicrobial therapy. Eur J Clin Microbiol Infect Dis. (2017) 36:403–13. doi: 10.1007/s10096-016-2816-4 27812806 PMC5309286

[66] Goodswen SJ Kennedy PJ Ellis JT . A guide to current methodology and usage of reverse vaccinology towards in *silico* vaccine discovery. FEMS Microbiol Rev. (2023) 47:fuad004. doi: 10.1093/femsre/fuad004 36806618

[67] Connors J Cusimano G Mege N Woloszczuk K Konopka E Bell M . Using the power of innate immunoprofiling to understand vaccine design, infection, and immunity. Hum Vaccines Immunotherapeutics. (2023) 19:2267295. doi: 10.1080/21645515.2023.2267295 37885158 PMC10760375

[68] Shin G-W Palaksha KJ Kim Y-R Nho S-W Kim S Heo G-J . Application of immunoproteomics in developing a Streptococcus iniae vaccine for olive flounder (Paralichthys olivaceus). J Chromatogr B. (2007) 849:315–22. doi: 10.1016/j.jchromb.2006.11.009 17140864

[69] Sudheesh P LaFrentz B Call D Siems W LaPatra S Wiens G . Identification of potential vaccine target antigens by immunoproteomic analysis of a virulent and a non-virulent strain of the fish pathogen Flavobacterium psychrophilum. Dis Aquat Org. (2007) 74:37–47. doi: 10.3354/dao074037 17425262

[70] Dumetz F Duchaud E Claverol S Orieux N Papillon S Lapaillerie D . Analysis of the Flavobacterium psychrophilum outer-membrane subproteome and identification of new antigenic targets for vaccine by immunomics. Microbiology. (2008) 154:1793–801. doi: 10.1099/mic.0.2008/016600-0 18524934

[71] Li H Ye M-Z Peng B Wu H-K Xu C-X Xiong X-P . Immunoproteomic identification of polyvalent vaccine candidates from Vibrio parahaemolyticus outer membrane proteins. J Proteome Res. (2010) 9:2573–83. doi: 10.1021/pr1000219 20148598

[72] Wang N Yang Z Zang M Liu Y Lu C . Identification of Omp38 by immunoproteomic analysis and evaluation as a potential vaccine antigen against Aeromonas hydrophila in Chinese breams. Fish Shellfish Immunol. (2013) 34:74–81. doi: 10.1016/j.fsi.2012.10.003 23063539

[73] Peng B Ye J Han Y Zeng L Zhang J Li H . Identification of polyvalent protective immunogens from outer membrane proteins in Vibrio parahaemolyticus to protect fish against bacterial infection. Fish Shellfish Immunol. (2016) 54:204–10. doi: 10.1016/j.fsi.2016.04.012 27071519

[74] Xing J Xu H Wang Y Tang X Sheng X Zhan W . Identification of immunogenic proteins and evaluation of four recombinant proteins as potential vaccine antigens from Vibrio anguillarum in flounder (Paralichthys olivaceus). Vaccine. (2017) 35:3196–203. doi: 10.1016/j.vaccine.2017.04.071 28483195

[75] Sheng X Liu M Liu H Tang X Xing J Zhan W . Identification of immunogenic proteins and evaluation of recombinant PDHA1 and GAPDH as potential vaccine candidates against Streptococcus iniae infection in flounder (Paralichthys olivaceus). PloS One. (2018) 13:e0195450. doi: 10.1371/journal.pone.0195450 29847601 PMC5976140

[76] Sheng X Zhang H Liu M Tang X Xing J Chi H . Development and evaluation of recombinant B-cell multi-epitopes of PDHA1 and GAPDH as subunit vaccines against Streptococcus iniae infection in flounder (Paralichthys olivaceus). Vaccines. (2023) 11:624. doi: 10.3390/vaccines11030624 36992208 PMC10051852

[77] Jacobsen KL Griffin MJ Phinney BS Salemi M Yazdi Z Balami S . Use of shotgun immunoproteomics for the development of protein vaccines against Edwardsiella piscicida. Fish Shellfish Immunol. (2026) 168:110992. doi: 10.1016/j.fsi.2025.110992 41213346 PMC13542899

[78] Finlay BB Falkow S . Common themes in microbial pathogenicity revisited. Microbiol Mol Biol Rev. (1997) 61:136–69. doi: 10.1128/mmbr.61.2.136-169.1997 9184008 PMC232605

[79] Zubair M Khan FA Menghwar H Faisal M Ashraf M Rasheed MA . Progresses on bacterial secretomes enlighten research on Mycoplasma secretome. Microb Pathogen. (2020) 144:104160. doi: 10.1016/j.micpath.2020.104160 32194181

[80] Sharma A Sanduja P Anand A Mahajan P Guzman CA Yadav P . Advanced strategies for development of vaccines against human bacterial pathogens. World J Microbiol Biotechnol. (2021) 37:67. doi: 10.1007/s11274-021-03021-6 33748926 PMC7982316

[81] Bidmos FA Siris S Gladstone CA Langford PR . Bacterial vaccine antigen discovery in the reverse vaccinology 2.0 era: Progress and challenges. Front Immunol. (2018) 9:2315. doi: 10.3389/fimmu.2018.02315 30349542 PMC6187972

[82] Rappuoli R Bottomley MJ D’Oro U Finco O De Gregorio E . Reverse vaccinology 2.0: Human immunology instructs vaccine antigen design. J Exp Med. (2016) 213:469–81. doi: 10.1084/jem.20151960 27022144 PMC4821650

[83] Tammas I Bitchava K Gelasakis AI . Transforming aquaculture through vaccination: A review on recent developments and milestones. Vaccines. (2024) 12:732. doi: 10.3390/vaccines12070732 39066370 PMC11281524

[84] Merkley ED Sego LH Lin A Leiser OP Kaiser BLD Adkins JN . Protein abundances can distinguish between naturally-occurring and laboratory strains of Yersinia pestis, the causative agent of plague. PloS One. (2017) 12:e0183478. doi: 10.1371/journal.pone.0183478 28854255 PMC5576697

[85] Tan A Atack JM Jennings MP Seib KL . The capricious nature of bacterial pathogens: Phasevarions and vaccine development. Front Immunol. (2016) 7. doi: 10.3389/fimmu.2016.00586 28018352 PMC5149525

[86] Giefing C Meinke AL Hanner M Henics T Minh DB Gelbmann D . Discovery of a novel class of highly conserved vaccine antigens using genomic scale antigenic fingerprinting of pneumococcus with human antibodies. J Exp Med. (2008) 205:117–31. doi: 10.1084/jem.20071168 18166586 PMC2234372

[87] Coiras M Camafeita E López‐Huertas MR Calvo E López JA Alcamí J . Application of proteomics technology for analyzing the interactions between host cells and intracellular infectious agents. Proteomics. (2008) 8:852–73. doi: 10.1002/pmic.200700664 18297655 PMC7167661

[88] Escudero-Pérez B Lawrence P Castillo-Olivares J . Immune correlates of protection for SARS-CoV-2, Ebola and Nipah virus infection. Front Immunol. (2023) 14:1156758. doi: 10.3389/fimmu.2023.1156758 37153606 PMC10158532

[89] Bethe U Pana ZD Drosten C Goossens H König F Marchant A . Innovative approaches for vaccine trials as a key component of pandemic preparedness – a white paper. Infection. (2024) 52:2135–44. doi: 10.1007/s15010-024-02347-1 39017997 PMC11621139

[90] Muñoz-Atienza E Díaz-Rosales P Tafalla C . Systemic and mucosal B and T cell responses upon mucosal vaccination of teleost fish. Front Immunol. (2021) 11:622377. doi: 10.3389/fimmu.2020.622377 33664735 PMC7921309

[91] Du Y Hu X Miao L Chen J . Current status and development prospects of aquatic vaccines. Front Immunol. (2022) 13:1040336. doi: 10.3389/fimmu.2022.1040336 36439092 PMC9684733

[92] Secombes CJ . Fish immunity: The potential impact on vaccine development and performance: Fish immunity. Aquacult Res. (2011) 42:90–2. doi: 10.1111/j.1365-2109.2010.02673.x40046247

[93] De Pascalis R Chou AY Bosio CM Huang C-Y Follmann DA Elkins KL . Development of functional and molecular correlates of vaccine-induced protection for a model intracellular pathogen, F. tularensis LVS. PloS Pathog. (2012) 8:e1002494. doi: 10.1371/journal.ppat.1002494 22275868 PMC3262015

[94] Zhou Q Chen Y Chen Z Wang L Ma X Wang J . Genomics and transcriptomics reveal new molecular mechanism of vibriosis resistance in fish. Front Immunol. (2022) 13:974604. doi: 10.3389/fimmu.2022.974604 36304468 PMC9592550

[95] Li J-N Zhao Y-T Cao S-L Wang H Zhang J-J . Integrated transcriptomic and proteomic analyses of grass carp intestines after vaccination with a double-targeted DNA vaccine of Vibrio mimicus. Fish Shellfish Immunol. (2020) 98:641–52. doi: 10.1016/j.fsi.2019.10.045 31678536

[96] Puente-Marín S Nombela I Chico V Ciordia S Mena MC Perez L . Potential role of rainbow trout erythrocytes as mediators in the immune response induced by a DNA vaccine in fish. Vaccines. (2019) 7:60. doi: 10.3390/vaccines7030060 31277329 PMC6789471

[97] Puente-Marín S Nombela I Chico V Ciordia S Mena MC Coll J . Rainbow trout erythrocytes ex vivo transfection with a DNA vaccine encoding VHSV glycoprotein G induces an antiviral immune response. Front Immunol. (2018) 9:2477. doi: 10.3389/fimmu.2018.02477 30429850 PMC6220650

[98] Carvalho I Schoninger FB Cunha A Peixoto D Brito F Simões L . Immunomodulatory effects of dietary methionine supplementation in rainbow trout (Oncorhynchus mykiss) juveniles: Insights following vaccination and infection response against Yersinia ruckeri. Front Immunol. (2025) 16:1706922. doi: 10.3389/fimmu.2025.1706922 41256851 PMC12620199

[99] Hoare R Shahin K McLean K Adams A Thompson KD . Skin mucus proteins of rainbow trout (*Oncorhynchus mykiss*) in response to mucosal vaccination and challenge with *Flavobacterium psychrophilum*. J Fish Dis. (2022) 45:491–5. doi: 10.1111/jfd.13562 34905629 PMC9299601

[100] Shi X Jiang L Ye M Yanglang A Mikkelborg MK Tang X . Peritoneal exosomes mediate activation of CD4+ T cells and enhancement of vaccine efficacy in teleost. Cell Commun Signal. (2026) 24:201. doi: 10.1186/s12964-026-02752-z 41721417 PMC13032352

[101] Ricklin D Reis ES Mastellos DC Gros P Lambris JD . Complement component C3 – the “Swiss Army Knife” of innate immunity and host defense. Immunol Rev. (2016) 274:33–58. doi: 10.1111/imr.12500 27782325 PMC5427221

[102] Wu M Jia B Li M . Complement C3 and activated fragment C3a are involved in complement activation and anti-bacterial immunity. Front Immunol. (2022) 13:813173. doi: 10.3389/fimmu.2022.813173 35281048 PMC8913944

[103] Turner NA Moake JL . The specific contributions of factor H and factor I in controlling fluid phase activation of the alternative complement pathway. Sci Rep. (2025) 15:10883. doi: 10.1038/s41598-025-95533-6 40158007 PMC11954907

[104] Brandwijk RJMGE Michels MAHM Van Rossum M De Nooijer AH Nilsson PH De Bruin WCC . Pitfalls in complement analysis: a systematic literature review of assessing complement activation. Front Immunol. (2022) 13:1007102. doi: 10.3389/fimmu.2022.1007102 36330514 PMC9623276

[105] Zwarthoff SA Berends ETM Mol S Ruyken M Aerts PC Józsi M . Functional characterization of alternative and classical pathway C3/C5 convertase activity and inhibition using purified models. Front Immunol. (2018) 9:1691. doi: 10.3389/fimmu.2018.01691 30083158 PMC6064732

[106] Forn-Cuní G Reis ES Dios S Posada D Lambris JD Figueras A . The evolution and appearance of C3 duplications in fish originate an exclusive teleost c3 gene form with anti-inflammatory activity. PloS One. (2014) 9:e99673. doi: 10.1371/journal.pone.0099673 24926798 PMC4057122

[107] Holland MCH Lambris JD . The complement system in teleosts. Fish Shellfish Immunol. (2002) 12:399–420. doi: 10.1006/fsim.2001.0408 12194452

[108] Li M Zhang H . An overview of complement systems in teleosts. Dev Comp Immunol. (2022) 137:104520. doi: 10.1016/j.dci.2022.104520 36041641

[109] Salinas I . The mucosal immune system of teleost fish. Biology. (2015) 4:525–39. doi: 10.3390/biology4030525 26274978 PMC4588148

[110] Yu Y Wang Q Huang Z Ding L Xu Z . Immunoglobulins, mucosal immunity and vaccination in teleost fish. Front Immunol. (2020) 11:567941. doi: 10.3389/fimmu.2020.567941 33123139 PMC7566178

[111] Li S Rouphael N Duraisingham S Romero-Steiner S Presnell S Davis C . Molecular signatures of antibody responses derived from a systems biology study of five human vaccines. Nat Immunol. (2014) 15:195–204. doi: 10.1038/ni.2789 24336226 PMC3946932

[112] Querec TD Akondy RS Lee EK Cao W Nakaya HI Teuwen D . Systems biology approach predicts immunogenicity of the yellow fever vaccine in humans. Nat Immunol. (2009) 10:116–25. doi: 10.1038/ni.1688 19029902 PMC4049462

[113] Carr SA Abbatiello SE Ackermann BL Borchers C Domon B Deutsch EW . Targeted peptide measurements in biology and medicine: best practices for mass spectrometry-based assay development using a fit-for-purpose approach. Mol Cell Proteomics. (2014) 13:907–17. doi: 10.1074/mcp.M113.036095 24443746 PMC3945918

[114] Picotti P Aebersold R . Selected reaction monitoring–based proteomics: workflows, potential, pitfalls and future directions. Nat Methods. (2012) 9:555–66. doi: 10.1038/nmeth.2015 22669653

[115] Medzhitov R Schneider DS Soares MP . Disease tolerance as a defense strategy. Science. (2012) 335:936–41. doi: 10.1126/science.1214935 22363001 PMC3564547

[116] Schneider DS Ayres JS . Two ways to survive infection: what resistance and tolerance can teach us about treating infectious diseases. Nat Rev Immunol. (2008) 8:889–95. doi: 10.1038/nri2432 18927577 PMC4368196

[117] Jones DR Rutan BJ Wargo AR . Impact of vaccination and pathogen exposure dosage on shedding kinetics of infectious hematopoietic necrosis virus (IHNV) in rainbow trout. J Aquat Anim Health. (2020) 32:95–108. doi: 10.1002/aah.10108 32443164 PMC7540492

[118] Oberg AL Vitek O . Statistical design of quantitative mass spectrometry-based proteomic experiments. J Proteome Res. (2009) 8:2144–56. doi: 10.1021/pr8010099 19222236

[119] Percie Du Sert N Hurst V Ahluwalia A Alam S Avey MT Baker M . The ARRIVE guidelines 2.0: updated guidelines for reporting animal research. PloS Biol. (2020) 18:e3000410. doi: 10.1371/journal.pbio.3000410 32663219 PMC7360023

[120] Taylor CF Paton NW Lilley KS Binz P-A Julian RK Jones AR . The minimum information about a proteomics experiment (MIAPE). Nat Biotechnol. (2007) 25:887–93. doi: 10.1038/nbt1329 17687369

[121] Jaiswal S Rasal KD Chandra T Prabha R Iquebal MA Rai A . Proteomics in fish health and aquaculture productivity management: status and future perspectives. Aquaculture. (2023) 566:739159. doi: 10.1016/j.aquaculture.2022.73915942574925

[122] Nesvizhskii AI Aebersold R . Interpretation of shotgun proteomic data. Mol Cell Proteomics. (2005) 4:1419–40. doi: 10.1074/mcp.R500012-MCP200 16009968

[123] Glasauer SMK Neuhauss SCF . Whole-genome duplication in teleost fishes and its evolutionary consequences. Mol Genet Genomics. (2014) 289:1045–60. doi: 10.1007/s00438-014-0889-2 25092473

[124] Jaillon O Aury J-M Brunet F Petit J-L Stange-Thomann N Mauceli E . Genome duplication in the teleost fish Tetraodon nigroviridis reveals the early vertebrate proto-karyotype. Nature. (2004) 431:946–57. doi: 10.1038/nature03025 15496914

[125] Kelkar DS Provost E Chaerkady R Muthusamy B Manda SS Subbannayya T . Annotation of the zebrafish genome through an integrated transcriptomic and proteomic analysis. Mol Cell Proteomics. (2014) 13:3184–98. doi: 10.1074/mcp.M114.038299 25060758 PMC4223501

[126] Nesvizhskii AI . Proteogenomics: concepts, applications and computational strategies. Nat Methods. (2014) 11:1114–25. doi: 10.1038/nmeth.3144 25357241 PMC4392723

[127] Munjal A Rex DAB Garg P Prasad TSK Mishra SK Malhotra Y . Mass spectrometric and artificial intelligence‐based identification of the secretome of *Plasmodium falciparum* merozoites to provide novel candidates for vaccine development pipeline. Proteomics Clin Apps. (2024) 18:e202300115. doi: 10.1002/prca.202300115 39082488

[128] Nakaya HI Wrammert J Lee EK Racioppi L Marie-Kunze S Haining WN . Systems biology of vaccination for seasonal influenza in humans. Nat Immunol. (2011) 12:786–95. doi: 10.1038/ni.2067 21743478 PMC3140559

[129] Tsang JS Schwartzberg PL Kotliarov Y Biancotto A Xie Z Germain RN . Global analyses of human immune variation reveal baseline predictors of postvaccination responses. Cell. (2014) 157:499–513. doi: 10.1016/j.cell.2014.03.031 24725414 PMC4139290

[130] Wang Y Zhu Q Sun R Yi X Huang L Hu Y . Longitudinal proteomic investigation of COVID-19 vaccination. Protein Cell. (2023) 14:668–82. doi: 10.1093/procel/pwad004 36930526 PMC10501184

[131] Bowden TJ . Modulation of the immune system of fish by their environment. Fish Shellfish Immunol. (2008) 25:373–83. doi: 10.1016/j.fsi.2008.03.017 18562213

[132] Mischak H Allmaier G Apweiler R Attwood T Baumann M Benigni A . Recommendations for biomarker identification and qualification in clinical proteomics. Sci Transl Med. (2010) 2:46ps42. doi: 10.1126/scitranslmed.3001249 20739680

[133] Nakayasu ES Gritsenko M Piehowski PD Gao Y Orton DJ Schepmoes AA . Tutorial: best practices and considerations for mass-spectrometry-based protein biomarker discovery and validation. Nat Protoc. (2021) 16:3737–60. doi: 10.1038/s41596-021-00566-6 34244696 PMC8830262

[134] Thorarensen H Kubiriza GK Imsland AK . Experimental design and statistical analyses of fish growth studies. Aquaculture. (2015) 448:483–90. doi: 10.1016/j.aquaculture.2015.05.01842574925

[135] Peterson AC Russell JD Bailey DJ Westphall MS Coon JJ . Parallel reaction monitoring for high resolution and high mass accuracy quantitative, targeted proteomics. Mol Cell Proteomics. (2012) 11:1475–88. doi: 10.1074/mcp.O112.020131 22865924 PMC3494192

[136] Gerber SA Rush J Stemman O Kirschner MW Gygi SP . Absolute quantification of proteins and phosphoproteins from cell lysates by tandem MS. Proc Natl Acad Sci USA. (2003) 100:6940–5. doi: 10.1073/pnas.0832254100 12771378 PMC165809

[137] Addona TA Abbatiello SE Schilling B Skates SJ Mani DR Bunk DM . Multi-site assessment of the precision and reproducibility of multiple reaction monitoring–based measurements of proteins in plasma. Nat Biotechnol. (2009) 27:633–46. doi: 10.1038/nbt.1546 19561596 PMC2855883

[138] Sandve GK Nekrutenko A Taylor J Hovig E . Ten simple rules for reproducible computational research. PloS Comput Biol. (2013) 9:e1003285. doi: 10.1371/journal.pcbi.1003285 24204232 PMC3812051

[139] Lazar C Gatto L Ferro M Bruley C Burger T . Accounting for the multiple natures of missing values in label-free quantitative proteomics data sets to compare imputation strategies. J Proteome Res. (2016) 15:1116–25. doi: 10.1021/acs.jproteome.5b00981 26906401

[140] Deutsch EW Bandeira N Perez-Riverol Y Sharma V Carver JJ Mendoza L . The ProteomeXchange consortium in 2026: making proteomics data FAIR. Nucleic Acids Res. (2026) 54:D459–69. doi: 10.1093/nar/gkaf1146 41206473 PMC12807779

[141] Vizcaíno JA Foster JM Martens L . Proteomics data repositories: providing a safe haven for your data and acting as a springboard for further research. J Proteomics. (2010) 73:2136–46. doi: 10.1016/j.jprot.2010.06.008 20615486 PMC2958306

[142] Purcell AW Ramarathinam SH Ternette N . Mass spectrometry–based identification of MHC-bound peptides for immunopeptidomics. Nat Protoc. (2019) 14:1687–707. doi: 10.1038/s41596-019-0133-y 31092913

[143] Pulendran B Li S Nakaya HI . Systems vaccinology. Immunity. (2010) 33:516–29. doi: 10.1016/j.immuni.2010.10.006 21029962 PMC3001343

[144] Li S Sullivan NL Rouphael N Yu T Banton S Maddur MS . Metabolic phenotypes of response to vaccination in humans. Cell. (2017) 169:862–877.e17. doi: 10.1016/j.cell.2017.04.026 28502771 PMC5711477

[145] Nakaya HI Hagan T Duraisingham SS Lee EK Kwissa M Rouphael N . Systems analysis of immunity to influenza vaccination across multiple years and in diverse populations reveals shared molecular signatures. Immunity. (2015) 43:1186–98. doi: 10.1016/j.immuni.2015.11.012 26682988 PMC4859820

[146] Natnan M Mayalvanan Y Jazamuddin F Aizat W Low C-F Goh H-H . Omics strategies in current advancements of infectious fish disease management. Biology. (2021) 10:1086. doi: 10.3390/biology10111086 34827079 PMC8614662

[147] Schoof EM Furtwängler B Üresin N Rapin N Savickas S Gentil C . Quantitative single-cell proteomics as a tool to characterize cellular hierarchies. Nat Commun. (2021) 12:3341. doi: 10.1038/s41467-021-23667-y 34099695 PMC8185083

[148] Specht H Emmott E Petelski AA Huffman RG Perlman DH Serra M . Single-cell proteomic and transcriptomic analysis of macrophage heterogeneity using SCoPE2. Genome Biol. (2021) 22:50. doi: 10.1186/s13059-021-02267-5 33504367 PMC7839219

[149] Andresen AMS Taylor RS Grimholt U Daniels RR Sun J Dobie R . Mapping the cellular landscape of Atlantic salmon head kidney by single cell and single nucleus transcriptomics. Fish Shellfish Immunol. (2024) 146:109357. doi: 10.1016/j.fsi.2024.109357 38181891

[150] Chan JTH Kadri S Köllner B Rebl A Korytář T . RNA-Seq of single fish cells – seeking out the leukocytes mediating immunity in teleost fishes. Front Immunol. (2022) 13:798712. doi: 10.3389/fimmu.2022.798712 35140719 PMC8818700

[151] Lundberg E Borner GHH . Spatial proteomics: a powerful discovery tool for cell biology. Nat Rev Mol Cell Biol. (2019) 20:285–302. doi: 10.1038/s41580-018-0094-y 30659282

[152] Mund A Coscia F Kriston A Hollandi R Kovács F Brunner A-D . Deep visual proteomics defines single-cell identity and heterogeneity. Nat Biotechnol. (2022) 40:1231–40. doi: 10.1038/s41587-022-01302-5 35590073 PMC9371970

[153] Olsen JV Blagoev B Gnad F Macek B Kumar C Mortensen P . Global, *in vivo*, and site-specific phosphorylation dynamics in signaling networks. Cell. (2006) 127:635–48. doi: 10.1016/j.cell.2006.09.026 17081983

[154] Moremen KW Tiemeyer M Nairn AV . Vertebrate protein glycosylation: diversity, synthesis and function. Nat Rev Mol Cell Biol. (2012) 13:448–62. doi: 10.1038/nrm3383 22722607 PMC3934011

[155] Swatek KN Komander D . Ubiquitin modifications. Cell Res. (2016) 26:399–422. doi: 10.1038/cr.2016.39 27012465 PMC4822133

[156] Kleifeld O Doucet A Auf Dem Keller U Prudova A Schilling O Kainthan RK . Isotopic labeling of terminal amines in complex samples identifies protein N-termini and protease cleavage products. Nat Biotechnol. (2010) 28:281–8. doi: 10.1038/nbt.1611 20208520

[157] Chalkley RJ Clauser KR . Modification site localization scoring: strategies and performance. Mol Cell Proteomics. (2012) 11:3–14. doi: 10.1074/mcp.R111.015305 22328712 PMC3418845

[158] Greener JG Kandathil SM Moffat L Jones DT . A guide to machine learning for biologists. Nat Rev Mol Cell Biol. (2022) 23:40–55. doi: 10.1038/s41580-021-00407-0 34518686

[159] Vabalas A Gowen E Poliakoff E Casson AJ . Machine learning algorithm validation with a limited sample size. PloS One. (2019) 14:e0224365. doi: 10.1371/journal.pone.0224365 31697686 PMC6837442

[160] Varma S Simon R . Bias in error estimation when using cross-validation for model selection. BMC Bioinf. (2006) 7:91. doi: 10.1186/1471-2105-7-91 16504092 PMC1397873

[161] Figueroa C Veloso P Espin L Dixon B Torrealba D Elalfy IS . Host genetic variation explains reduced protection of commercial vaccines against Piscirickettsia salmonis in Atlantic salmon. Sci Rep. (2020) 10:18252. doi: 10.1038/s41598-020-70847-9 33106499 PMC7588420

[162] Van Der Maas L Danial M Kersten GFA Metz B Meiring HD . Mass spectrometry-based quantification of the antigens in aluminum hydroxide-adjuvanted diphtheria-tetanus-acellular-pertussis combination vaccines. Vaccines. (2022) 10:1078. doi: 10.3390/vaccines10071078 35891242 PMC9323524

[163] Midtlyng PJ Hendriksen C Balks E Bruckner L Elsken L Evensen Ø . Three Rs approaches in the production and quality control of fish vaccines. Biologicals. (2011) 39:117–28. doi: 10.1016/j.biologicals.2011.02.001 21371907

