# Supplementary material for: Proteomic signatures of vaccine-induced immunity in teleost fish: from antigen discovery to correlates of protection
Source: Front Immunol. 2026 Oct 26;17:1965383. doi: 10.3389/fimmu.2026.1965383 (PMC13746870; doi:10.3389/fimmu.2026.1965383)
Supplement: Supplementary file 1 [file Table1.docx]

| **Study** | **Fish model and vaccine** | **Sample and timing** | **Proteomic design** | **Principal protein / pathway findings** | **Protection or outcome linkage** | **Principal limitation** |
| --- | --- | --- | --- | --- | --- | --- |
| (Bakke et al. 2020) | Rainbow trout; hen egg lysozyme in complete Freund’s adjuvant | Plasma; longitudinal days 0–84 | Label-free LC–MS/MS; repeated measures in six immunized fish plus adjuvant control | Complement, acute-phase and lipoprotein-associated dynamics | No protective pathogen challenge; no later-outcome prediction | Model antigen, very small cohort and limited control structure |
| (Puente-Marín et al. 2018) | Rainbow trout erythrocytes; ex vivo VHSV glycoprotein-G DNA transfection | Erythrocytes/conditioned medium; day 6 ex vivo | Pooled ex vivo proteomics with antiviral functional assay | Antigen presentation, proteasome, ISG15 and redox/ROS-detoxification responses | Conditioned medium reduced VHSV infection in a cell line; no vaccinated-fish challenge outcome | Ex vivo pooled model; cannot establish an in vivo correlate of protection |
| (Puente-Marín et al. 2019) | Rainbow trout; DNA vaccine encoding VHSV glycoprotein G | Peripheral-blood erythrocytes after in vivo immunization | Integrated transcriptomic and proteomic profiling | MHC-I antigen processing/presentation, Fc-receptor signalling, autophagy and innate response | Specific antibody induction supported immune activation; profiles were not tested as individual predictors | Mechanistic immune-response study without independent predictive validation |
| (Hoare et al. 2022) | Rainbow trout; polyvalent immersion vaccine against F. psychrophilum | Skin mucus before challenge and 32 days after challenge | Mucus proteomics across vaccination/challenge conditions | Vaccination- and challenge-associated mucosal proteins, including annexin-related responses | Group-level vaccine/challenge context; protein profiles not linked prospectively to individual outcomes | Post-challenge measurements confound vaccine response with infection and recovery |
| (Chérif et al. 2025) | European sea bass; chemically inactivated nodavirus vaccine | Serum; vaccinated versus unvaccinated cohorts | Shotgun LC–MS/MS serological profiling | Antioxidant, coagulation and immune-associated changes | No pre-challenge profile-to-individual-outcome linkage | Small separate cohorts; exploratory single-study signature |
| (Carvalho et al. 2025) | Rainbow trout; methionine diet × commercial Yersinia ruckeri vaccine | Plasma at 21 days post-vaccination and early post-infection time points | Proteomics embedded in a factorial nutrition–vaccination–challenge study | Post-infection haemostasis-related and treatment-specific responses | Mortality, bacterial burden and proteomics were compared at group level; strongest proteomic effects were post-challenge | Diet, vaccination and infection effects are intertwined; no individual pre-challenge prediction |
| (Shi et al. 2026) | Olive flounder; inactivated V. anguillarum vaccine and peritoneal-cell exosomes | Peritoneal exosomes and immune cells | Multi-omic exosome profiling plus depletion/functional experiments | MHC-II and CD80/86-positive exosome subpopulations supported CD4+ T-cell activation and vaccine enhancement | Functional evidence for an immune mechanism, not a transferable pre-challenge biomarker | Mechanistic evidence does not establish a validated correlate of protection; independent transfer not shown |
| (Li et al. 2020) | Grass carp; oral double-targeted DNA vaccine against Vibrio mimicus | Intestine; vaccinated versus unvaccinated fish. | RNA-seq integrated with iTRAQ-coupled LC–MS/MS; selected DEPs confirmed by MRM | 1,173 DEPs; 250 matched DEPs/DEGs; immune-related enrichment involving phagosome, antigen processing and presentation, complement/coagulation, NLR and MAPK pathways | Vaccine-responsive/immune-activation profile. MRM confirmed selected protein measurements, but no individual pre-challenge prediction was tested. | Group-level vaccinated-versus-control comparison; no independent predictive validation |

1. Bakke, Fiona K., Milena M. Monte, David A. Stead, Dwight R. Causey, Alex Douglas, Daniel J. Macqueen, and Helen Dooley. 2020. “Plasma Proteome Responses in Salmonid Fish Following Immunization.” *Frontiers in Immunology* 11: 581070. doi:10.3389/fimmu.2020.581070.
2. Carvalho, Inês, Felipe Bolgenhagen Schoninger, André Cunha, Diogo Peixoto, Francisca Brito, Luara Simões, Mariana Vaz, et al. 2025. “Immunomodulatory Effects of Dietary Methionine Supplementation in Rainbow Trout (Oncorhynchus Mykiss) Juveniles: Insights Following Vaccination and Infection Response against Yersinia Ruckeri.” *Frontiers in Immunology* 16: 1706922. doi:10.3389/fimmu.2025.1706922.
3. Chérif, Nadia, Kais Ghedira, Houda Agrebi, Semah Najahi, Hiba Mejri, Saifeddine Azouz, Mélodie Kielbasa, et al. 2025. “Proteomic Profiling of the Serological Response to a Chemically-Inactivated Nodavirus Vaccine in European Sea Bass Dicentrarchus Labrax.” *Veterinary Research Communications* 49(3): 125. doi:10.1007/s11259-025-10688-x.
4. Hoare, R., K. Shahin, K. McLean, A. Adams, and K. D. Thompson. 2022. “Skin Mucus Proteins of Rainbow Trout ( *Oncorhynchus Mykiss* ) in Response to Mucosal Vaccination and Challenge with *Flavobacterium Psychrophilum*.” *Journal of Fish Diseases* 45(3): 491–95. doi:10.1111/jfd.13562.
5. Li, Jin-Nian, Yu-Ting Zhao, Shou-Lin Cao, Hong Wang, and Jia-Jun Zhang. 2020. “Integrated Transcriptomic and Proteomic Analyses of Grass Carp Intestines after Vaccination with a Double-Targeted DNA Vaccine of Vibrio Mimicus.” *Fish & Shellfish Immunology* 98: 641–52. doi:10.1016/j.fsi.2019.10.045.
6. Puente-Marín, Sara, Ivan Nombela, Veronica Chico, Sergio Ciordia, Maria Carmen Mena, Julio Coll, Luis Mercado, and Maria Del Mar Ortega-Villaizan. 2018. “Rainbow Trout Erythrocytes Ex Vivo Transfection With a DNA Vaccine Encoding VHSV Glycoprotein G Induces an Antiviral Immune Response.” *Frontiers in Immunology* 9: 2477. doi:10.3389/fimmu.2018.02477.
7. Puente-Marín, Sara, Ivan Nombela, Veronica Chico, Sergio Ciordia, Maria Carmen Mena, Luis Perez, Julio Coll, and Maria Del Mar Ortega-Villaizan. 2019. “Potential Role of Rainbow Trout Erythrocytes as Mediators in the Immune Response Induced by a DNA Vaccine in Fish.” *Vaccines* 7(3): 60. doi:10.3390/vaccines7030060.
8. Shi, Xueyan, Lixia Jiang, Miao Ye, Arat Yanglang, Marie Kristin Mikkelborg, Xiaoqian Tang, Jing Xing, et al. 2026. “Peritoneal Exosomes Mediate Activation of CD4+ T Cells and Enhancement of Vaccine Efficacy in Teleost.” *Cell Communication and Signaling* 24(1): 201. doi:10.1186/s12964-026-02752-z.
