# Supplementary material for: Proteomic signatures of vaccine-induced immunity in teleost fish: from antigen discovery to correlates of protection
Source: Front Immunol. 2026 Oct 26;17:1965383. doi: 10.3389/fimmu.2026.1965383 (PMC13746870; doi:10.3389/fimmu.2026.1965383)
Supplement: Supplementary file 2 [file Table2.docx]

**Table S3.** Additional searches for handpicked research articles/gaps/exploration during writing process

| **Platform** | **Exact query or prompt** | **Purpose** |
| --- | --- | --- |
| Google Scholar | "Fish immunology" AND "vaccinology" AND "proteomics" AND (review OR "research gaps" OR "future directions") | Exploratory reviews/gaps |
| Google Scholar | "immunoproteomics" AND "fish" AND ("vaccine development" OR "vaccinology") | Antigen discovery |
| Google Scholar | "Fish immunology" AND "proteomics" AND (challenges OR gaps OR "future perspectives") | Contextual literature |
| Google Scholar | ("proteomic signatures" OR proteomics) AND (vaccine OR "vaccine-induced immunity") AND ("teleost fish" OR fish) AND ("antigen discovery" OR "correlates of protection") | Core topic |
| Google Scholar | (proteomics OR proteomic) AND ("antigen discovery" OR "correlates of protection" OR "biomarkers of protection") AND vaccine AND (teleost OR fish) | Core topic |
| SciSpace | What are the latest review papers and research gaps in fish immunology, vaccinology, and proteomics? | Exploratory (n=20) |
| SciSpace | How is immunoproteomics used for fish vaccine development and what are the future directions? | Exploratory (n=20) |
