# Supplementary material for: Proteomic signatures of vaccine-induced immunity in teleost fish: from antigen discovery to correlates of protection
Source: Front Immunol. 2026 Oct 26;17:1965383. doi: 10.3389/fimmu.2026.1965383 (PMC13746870; doi:10.3389/fimmu.2026.1965383)
Supplement: Supplementary file 3 [file Table3.docx]

| **Study** | **Fish model** | **Pathogen** | **Proteomic / immunoproteomic workflow** | **Lead antigen(s)** | **Validation design** | **Candidate-specific evidence** | **Principal limitation** |
| --- | --- | --- | --- | --- | --- | --- | --- |
| (Z. Chen et al. 2004) | Carp | *A. hydrophila* | OMP fraction; 2-DE, immunoblot and MS; challenge-based screening | Several dominant immunogens; p6 strongest | Recombinant/candidate immunization followed by virulent challenge | 71.4% protection; activity also reported against *A. sobria* | Further experiment on independent cohort or field validation |
| (Shin et al. 2007) | Olive flounder | *S. iniae* | Whole-cell lysate and extracellular products; 2-DE immunoblot and MS | ECPs showed stronger immunoreactivity than whole-cell proteins | Formalin-killed cells with concentrated ECPs evaluated | Protective formulation, but protection not assigned to an individual MS-identified protein, information is limited. | Formulation-level information, rather than candidate-specific evidence is limited |
| (Sudheesh et al. 2007) | Rainbow  trout | *F. psychrophilum* | Virulent/non-virulent comparative proteomics; cellular/ECP immunoproteomics | HSP60, HSP70, ATP synthase, thermolysin and other immunogens | Immune recognition and strain comparison | No candidate-specific vaccination–challenge test | Antigenicity and expression do not establish protection |
| (Dumetz et al. 2008) | Rainbow trout | *F. psychrophilum* | Outer-membrane enrichment; 2-DE, LC–MS/MS and convalescent-serum immunoblotting | 25 immunoreactive proteins including OmpA, OmpH/P18 and FspA | Immune recognition in convalescent fish | No candidate-specific vaccination–challenge test | Candidate list requires expression/accessibility and protection testing |
| (Li et al. 2010) | Crucian carp | *V. parahaemolyticus* | Heterologous-antiserum OMP immunoproteomics | VPA1435, VP0764, VPA1186, VP1061 and VP2850 | Candidate immunization with homologous and heterologous challenges | VP1061/VP2850 protection and cross-protection against V. alginolyticus, *A. hydrophila* and *P. fluorescens* | Transfer across independent cohorts, production settings and routes not demonstrated |
| (Wang et al. 2013) | Chinese bream | *A. hydrophila* | OMP immunoproteomics followed by recombinant-antigen evaluation | Seven immunogens; Omp38 prioritized | Recombinant Omp38 immunization, immune assays and challenge | Significant candidate-specific protection | Single species–pathogen–laboratory context |
| (Peng et al. 2016) | Zebrafish | *V. parahaemolyticus* | OMP immunogen screening with active and passive evaluation | VP2309, VP0887, VPA0548 and VP1019 | Candidate immunization plus homologous/heterologous challenges | Homologous protection and varying cross-protection against A. hydrophila and P. fluorescens | Cross-protection varied by antigen and pathogen; no external replication |
| (Xing et al. 2017) | Olive flounder | *V. anguillarum* | Whole-cell immunoblotting followed by MS and recombinant-antigen testing | VAA, GroEL, OmpU and SpK | Recombinant proteins, immune phenotyping and challenge | RPS: VAA 78.38%, GroEL 72.97%, OmpU 64.86%, SpK 21.62% | Marked variation among candidates; same research setting requires external replication |
| (Sheng et al. 2018) | Olive flounder | *S. iniae* | Whole-cell immunoblotting and MS | PDHA1, BMP, LDH, OCT, LOx and GAPDH; PDHA1/GAPDH tested | Recombinant-antigen immunization and challenge | RPS: PDHA1 72.73%, GAPDH 63.64%, formalin-killed cells 36.36% | Promising candidate protection but no independent laboratory validation |
| (J. Chen et al. 2019) | Hybrid snakehead | *N. seriolae* with related *Nocardia spp.* | Comparative whole-cell immunoproteomics | DnaK, GroEL, RpsA, TerD, FHA, RplL and PspA | FHA-encoding DNA vaccine evaluated by challenge | 79.33% post-challenge survival reported for pcDNA-FHA | One candidate and one principal fish model; transferability remains unknown |
| (Sheng et al. 2023) | Olive flounder | *S. iniae* | B-cell multiepitope design derived from previously identified PDHA1/GAPDH antigens | Recombinant multiepitope derivatives | Immune-response and challenge evaluation | Follow-up protective refinement of the same antigen family | Follow-up within the same research programme; not independent external replication |
| (Jacobsen et al. 2026) | Rainbow trout/hybrid catfish sera; Chinook salmon validation | *E. piscicida* | Shotgun immunoproteomics; oral and intracoelomic candidate testing | 36 candidates; GroEL, GlyZip and COPIII tested | Candidate immunogenicity and challenge evaluation | Strong IgM responses; slight/non-significant survival for GlyZip/COPIII; GroEL anti-protective | Demonstrates that immunoreactivity may fail to predict protection |

**References**

1. Chen, Jianlin, Wanchun Tan, Wenji Wang, Suying Hou, Guoquan Chen, Liqun Xia, and Yishan Lu. 2019. “Identification of Common Antigens of Three Pathogenic Nocardia Species and Development of DNA Vaccine against Fish Nocardiosis.” *Fish & Shellfish Immunology* 95: 357–67. doi:10.1016/j.fsi.2019.09.038.
2. Chen, Zijun, Bo Peng, Sanying Wang, and Xuanxian Peng. 2004. “Rapid Screening of Highly Efficient Vaccine Candidates by Immunoproteomics.” *PROTEOMICS* 4(10): 3203–13. doi:10.1002/pmic.200300844.
3. Dumetz, Fabien, Eric Duchaud, Stéphane Claverol, Nicolas Orieux, Sandrine Papillon, Delphine Lapaillerie, and Michel Le Hénaff. 2008. “Analysis of the Flavobacterium Psychrophilum Outer-Membrane Subproteome and Identification of New Antigenic Targets for Vaccine by Immunomics.” *Microbiology* 154(6): 1793–1801. doi:10.1099/mic.0.2008/016600-0.
4. Jacobsen, Kim L., Matt J. Griffin, Brett S. Phinney, Michelle Salemi, Zeinab Yazdi, Sujita Balami, Caitlin E. Older, et al. 2026. “Use of Shotgun Immunoproteomics for the Development of Protein Vaccines against Edwardsiella Piscicida.” *Fish & Shellfish Immunology* 168: 110992. doi:10.1016/j.fsi.2025.110992.
5. Li, Hui, Ming-Zhi Ye, Bo Peng, Hong-Kai Wu, Chang-Xin Xu, Xiao-Peng Xiong, Chao Wang, San-Ying Wang, and Xuan-Xian Peng. 2010. “Immunoproteomic Identification of Polyvalent Vaccine Candidates from Vibrio Parahaemolyticus Outer Membrane Proteins.” *Journal of Proteome Research* 9(5): 2573–83. doi:10.1021/pr1000219.
6. Peng, Bo, Jin-zhou Ye, Yi Han, Li Zeng, Jian-ying Zhang, and Hui Li. 2016. “Identification of Polyvalent Protective Immunogens from Outer Membrane Proteins in Vibrio Parahaemolyticus to Protect Fish against Bacterial Infection.” *Fish & Shellfish Immunology* 54: 204–10. doi:10.1016/j.fsi.2016.04.012.
7. Sheng, Xiuzhen, Min Liu, Haibo Liu, Xiaoqian Tang, Jing Xing, and Wenbin Zhan. 2018. “Identification of Immunogenic Proteins and Evaluation of Recombinant PDHA1 and GAPDH as Potential Vaccine Candidates against Streptococcus Iniae Infection in Flounder (Paralichthys Olivaceus)” ed. Linsheng Song. *PLOS ONE* 13(5): e0195450. doi:10.1371/journal.pone.0195450.
8. Sheng, Xiuzhen, Honghua Zhang, Min Liu, Xiaoqian Tang, Jing Xing, Heng Chi, and Wenbin Zhan. 2023. “Development and Evaluation of Recombinant B-Cell Multi-Epitopes of PDHA1 and GAPDH as Subunit Vaccines against Streptococcus Iniae Infection in Flounder (Paralichthys Olivaceus).” *Vaccines* 11(3): 624. doi:10.3390/vaccines11030624.
9. Shin, Gee-Wook, K.J. Palaksha, Young-Rim Kim, Sung-Won Nho, Suk Kim, Gang-Joon Heo, Se-Chang Park, and Tae-Sung Jung. 2007. “Application of Immunoproteomics in Developing a Streptococcus Iniae Vaccine for Olive Flounder (Paralichthys Olivaceus).” *Journal of Chromatography B* 849(1–2): 315–22. doi:10.1016/j.jchromb.2006.11.009.
10. Sudheesh, Ps, Br LaFrentz, Dr Call, Wf Siems, Se LaPatra, Gd Wiens, and Kd Cain. 2007. “Identification of Potential Vaccine Target Antigens by Immunoproteomic Analysis of a Virulent and a Non-Virulent Strain of the Fish Pathogen Flavobacterium Psychrophilum.” *Diseases of Aquatic Organisms* 74: 37–47. doi:10.3354/dao074037.
11. Wang, Na, Zhao Yang, Mingfa Zang, Yongjie Liu, and Chengping Lu. 2013. “Identification of Omp38 by Immunoproteomic Analysis and Evaluation as a Potential Vaccine Antigen against Aeromonas Hydrophila in Chinese Breams.” *Fish & Shellfish Immunology* 34(1): 74–81. doi:10.1016/j.fsi.2012.10.003.
12. Xing, Jing, Hongsen Xu, Yang Wang, Xiaoqian Tang, Xiuzhen Sheng, and Wenbin Zhan. 2017. “Identification of Immunogenic Proteins and Evaluation of Four Recombinant Proteins as Potential Vaccine Antigens from Vibrio Anguillarum in Flounder ( Paralichthys Olivaceus ).” *Vaccine* 35(24): 3196–3203. doi:10.1016/j.vaccine.2017.04.071.
